# USP25 stabilises *GLI1* through K48‐linked deubiquitination within a reciprocal regulatory circuit in osteoarthritis models

**DOI:** 10.1002/ctm2.70851

**Published:** 2026-09-28

**Authors:** Qian Zhang, Yinyan Ye, Yijie Huang, Jun Li, Dongfeng Huang

**Affiliations:** ^1^ Department of Rehabilitation Medicine The Seventh Affiliated Hospital Sun Yat‐sen University Shenzhen China; ^2^ Department of Urology The Seventh Affiliated Hospital Sun Yat‐sen University Shenzhen China

**Keywords:** deubiquitination, *GLI1*, Hedgehog signalling, osteoarthritis, USP25

## Abstract

**Background:**

Osteoarthritis (OA) is characterised by progressive articular cartilage degeneration, yet effective disease‐modifying targets remain limited. The post‐translational mechanisms sustaining Hedgehog/*GLI1* signalling during OA progression remain incompletely understood.

**Methods:**

Bioinformatic analyses of GEO datasets were performed to evaluate *GLI1* expression across OA‐related tissue compartments. Independent cartilage datasets, paired human cartilage specimens, and primary human chondrocytes exposed to graded IL‐1β stimulation provided tissue‐ and cell‐relevant evidence. An unbiased deubiquitinase library screen combined with *GLI1*‐responsive luciferase assays was used to identify *GLI1*‐regulating deubiquitinases. The molecular function of USP25, its reciprocal regulation with *GLI1*, and the preclinical chondroprotective activity and target dependence of AZ1 were investigated in vitro and in vivo.

**Results:**

*GLI1* dysregulation in OA cartilage and primary human chondrocytes supported subsequent screening, which prioritised USP25 for mechanistic study. In paired cartilage from 24 OA patients, USP25 protein abundance was higher in grossly degenerated than in relatively preserved regions and showed a modest positive correlation with *GLI1*. USP25 directly interacted with *GLI1*, removed K48‐linked polyubiquitin chains, and limited its proteasomal degradation. Reciprocal gain‐ and loss‐of‐function and rescue experiments indicated that *GLI1* substantially, but not exclusively, mediated USP25‐associated inflammatory and cartilage‐catabolic phenotypes. *GLI1*, in turn, bound the *USP25* promoter and enhanced *USP25* transcription, establishing a reciprocal regulatory circuit. AZ1 showed preclinical chondroprotective activity in primary human chondrocytes and DMM mice. Residual AZ1 activity after USP25 silencing indicated partial USP25 dependence.

**Conclusions:**

These findings define a *GLI1*‐centred mechanistic branch of USP25 biology in OA‐relevant models and provide tissue‐level support for coordinated USP25‐*GLI1* expression in human OA cartilage. The reciprocal USP25‐*GLI1* circuit contributes to cartilage‐catabolic phenotypes, while AZ1 provides preclinical proof‐of‐concept as a pharmacological probe. Further studies are warranted to define target selectivity, long‐term safety, and clinical relevance.

## Graphical abstract

***GLI1*‐centred USP25‐*GLI1* reciprocal regulation in OA‐relevant models**. Hedgehog/*GLI1* signalling transcriptionally reinforces USP25 expression, while USP25 deubiquitinates and stabilises *GLI1*. This reciprocal circuit is associated with inflammatory and cartilage‐catabolic changes in the tested models. AZ1, a reported dual USP25/USP28 inhibitor, is presented as a proof‐of‐concept pharmacological probe; the AZ1‐associated phenotype observed in this study was partly USP25 dependent.

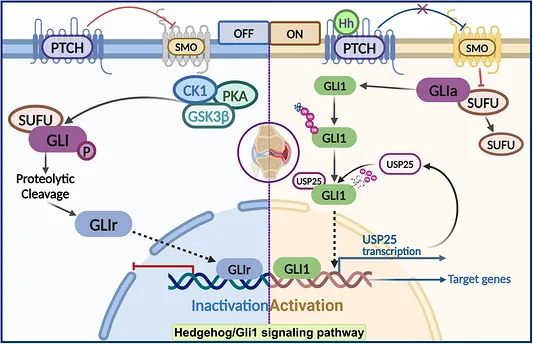

## 1 INTRODUCTION

Osteoarthritis (OA) is considered the most prevalent type of arthritis and the primary cause of long‐term disability, chronic pain, functional impairment, and reduced quality of life.^1^, ^2^, ^3^ The Global Burden of Disease Study 2021 showed that the number of people with OA is projected to approach 1 billion by 2050.^3^ Current management includes physical therapy, intra‐articular treatment, modification of risk factors, complementary approaches, and medial opening‐wedge high tibial osteotomy, while emerging strategies such as stem cell therapy and platelet‐rich plasma injection have also been explored.^4^, ^5^, ^6^, ^7^, ^8^ However, most available treatments primarily alleviate symptoms or preserve function and do not reliably prevent structural progression and reduce the complications. Therefore, identifying disease‐relevant molecular mechanisms and actionable therapeutic targets remains an important clinical need.

OA is a whole‐joint disorder involving articular cartilage degeneration, synovial inflammation, aberrant subchondral bone remodelling, mechanical imbalance, and metabolic disturbance.^9^, ^10^ Recent work further emphasises that OA is influenced by systemic metabolic and inflammatory comorbidities and organ crosstalk.^11^ At the joint level, multi‐compartment strategies targeting cartilage, synovium, and subchondral bone likewise underscore the interconnected nature of OA pathobiology.^12^ Among these changes, progressive chondrocyte dysfunction and loss of extracellular matrix (ECM) homeostasis are central to cartilage destruction. Persistent inflammatory stimulation enhances chondrocyte catabolism while suppressing matrix synthesis. Pro‐inflammatory cytokines, including IL‐1β, IL‐6, and TNF‐α, induce matrix metalloproteinases and ADAMTS family members, thereby accelerating ECM degradation and amplifying local inflammatory and mechanical stress.^13^, ^14^, ^15^, ^16^ Although numerous signalling pathways have been implicated in OA, many proposed targets have shown limited therapeutic efficacy, highlighting the need to define additional mechanisms that sustain cartilage‐catabolic signalling.^17^

Ubiquitination and deubiquitination are opposing post‐translational modifications that regulate protein stability, localisation, and activity. Increasing evidence indicates that dysregulated ubiquitin‐dependent signalling contributes to OA by altering inflammatory pathways and cartilage homeostasis.^18^, ^19^, ^20^ Several deubiquitinases (DUBs) have been implicated in OA‐related processes. USP25 is upregulated in IL‐1β‐treated mouse chondrocytes and has been reported to deubiquitinate thioredoxin‐interacting protein, thereby facilitating NLRP3 inflammasome activation and OA progression.^21^, ^22^ Thus, the present study does not seek to newly implicate USP25 in OA; instead, it asks whether USP25 engages an additional *GLI1*‐centred substrate and signalling branch relevant to cartilage‐catabolic phenotypes. Other DUBs regulate distinct pathogenic pathways: USP7 suppresses endoplasmic reticulum stress and NF‐κB signalling, USP14 enhances NF‐κB activation through IκBα degradation, USP3 and USP49 modulate TRAF6‐ and Wnt/β‐catenin‐related responses, and USP15 and UCH37 influence TGF‐β signalling and cartilage matrix synthesis.^23^, ^24^, ^25^, ^26^, ^27^, ^28^ These findings establish DUBs as important regulators of OA, but the relevant substrates and pathway‐specific functions of individual DUBs remain incompletely defined.

The Hedgehog pathway and its downstream transcription factor *GLI1* are implicated in chondrocyte dysfunction and cartilage degeneration. Because *GLI1* activity is influenced by protein turnover, deubiquitination may represent a critical mechanism for sustaining Hedgehog signalling. However, whether a specific DUB stabilises *GLI1* in OA, and whether reciprocal regulation between that DUB and *GLI1* contributes to disease progression, remain unclear. This knowledge gap also limits the development of pharmacological strategies targeting *GLI1* indirectly through its protein‐stability machinery.

In this study, we combined bioinformatic analyses, a DUB library screen, and *GLI1*‐responsive luciferase assays to identify USP25 as a deubiquitinase regulating *GLI1*. USP25 directly interacted with *GLI1*, removed K48‐linked polyubiquitin chains, and protected *GLI1* from proteasomal degradation. In turn, *GLI1* transcriptionally activated USP25, forming a reciprocal regulatory circuit associated with inflammatory and cartilage‐catabolic changes in OA‐relevant models. Mechanistically, the present study identifies a *GLI1*‐centred regulatory branch of USP25 signalling in OA‐relevant models. Intra‐articular AZ1 treatment was associated with attenuated Destabilization of the Medial Meniscus (DMM)‐associated cartilage pathology, and related protective effects were observed in OA‐like primary human chondrocytes. These findings support the use of AZ1 as a preclinical pharmacological probe while further defining the USP25‐*GLI1* regulatory mechanism in OA‐relevant models.

## 2 RESULTS

### 2.1 *GLI1* was nominated in a subchondral‐bone discovery dataset and supported in OA cartilage and chondrocytes

GSE51588 was used as a hypothesis‐generating discovery dataset. After normalisation, principal component analysis, and quality control, 35 OA and 6 normal subchondral‐bone samples were retained (Figure S1A). For Weighted Gene Co‐expression Network Analysis (WGCNA), a soft‐thresholding power of 20 was selected at a scale‐free topology fit of *R* ^2^ = 0.90 (Figure S1B and C). The average hierarchical clustering and dynamic tree clipping identified 14 modules, with the purple module exhibiting a strong correlation with OA progression (*r* = 0.62, *p* = 1.0 × 10^−5^) (Figure S1D). In addition, 151 hub genes exhibiting elevated expression levels in OA were identified based on a gene significance (GS) threshold of > 0.5 and module membership (MM) > 0.8. Differential‐expression analysis identified 1078 upregulated genes using |log_2_FC| > 1 and adjusted *p* < .05 (Figure S1E). A STRING protein‐protein interaction network yielded 229 upregulated genes with degree ≥ 10 (Figure S1F). Thirty‐six genes overlapped between the upregulated hub‐gene and interaction‐gene sets, and *GLI1* showed the largest increase among these candidates (Figure 1A and B). GSEA and *GLI1*‐based enrichment analyses indicated altered Hedgehog‐pathway‐related transcription in the discovery dataset (Figure 1C and D). Because GSE51588 profiles subchondral bone, these analyses were used only to nominate *GLI1* for follow‐up. Tissue relevance was then evaluated independently, beginning with cartilage datasets. After batch correction and integration of GSE178557, GSE129147, and GSE114007, dataset‐specific clustering was reduced (Figure S2A and B), and *GLI1* expression was higher in OA cartilage than in normal cartilage (*p* = 1.9 × 10^−5^; Figure S2C). Hedgehog‐pathway enrichment also differed between OA and normal cartilage (Figure S2D and E). In integrated synovial datasets (GSE1919, GSE12021, GSE55235, and GSE55457), *GLI1* expression was lower in OA synovium (*p* = 2.1 × 10^−5^; Figure S2F–H), with corresponding differences in Hedgehog‐pathway enrichment (Figure S2I and J). No significant difference in *GLI1* expression was detected in peripheral blood mononuclear cells from GSE300310 (*p* = .31), and neither enrichment analysis showed a significant Hedgehog‐pathway difference (Figure S2K–M). These divergent bulk‐tissue findings support compartment‐specific *GLI1* dysregulation, indicating that *GLI1* responses vary across tissue compartments and biological contexts.

**FIGURE 1:**
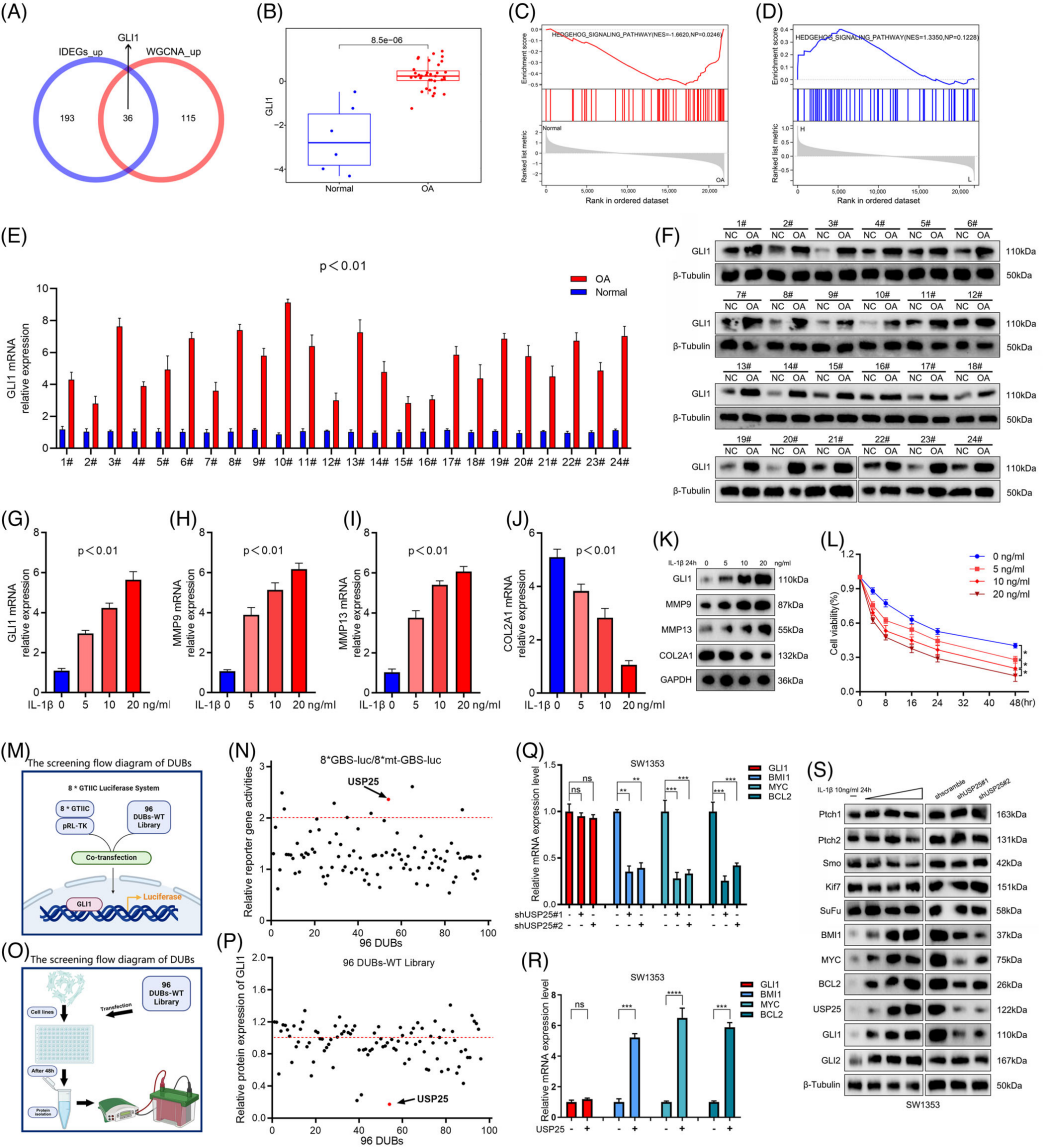
Cross‐tissue nomination of *GLI1* and prioritisation of USP25 as a *GLI1*‐regulating deubiquitinase in OA‐relevant models. (A) Venn diagram of IDEGs and hub genes. (B) *GLI1* expression level in the OA and normal groups in a GSE51588 dataset. (C) GSEA demonstrated that the Hedgehog pathway levels were markedly changed in OA relative to the normal group in GSE51588. (D) Single‐gene enrichment analysis revealed that *GLI1* mRNA expression level in OA samples of GSE51588 was notably changed. (E, F) *GLI1* mRNA (E) and protein (F) expression in paired grossly degenerated and relatively preserved cartilage selected by macroscopic appearance from the same OA joints of 24 patients. Statistical comparisons were performed using paired two‐tailed Student's *t*‐tests. (G–J) RT‐qPCR analysis of *GLI1* (G), *MMP9* (H), *MMP13* (I), and *COL2A1* (J) in primary human chondrocytes treated with 0, 5, 10, or 20 ng/mL IL‐1β for 24 h. (K) Immunoblotting of *GLI1*, MMP9, MMP13, and COL2A1 in primary human chondrocytes treated with the indicated IL‐1β concentrations for 24 h. (L) CCK‐8 analysis of cellular metabolic viability in primary human chondrocytes under the indicated IL‐1β concentrations and time points; treatment and time were analysed jointly by two‐way ANOVA with multiplicity‐adjusted post hoc comparisons. (M) Flow chart for DUB identification with the 8 × GTIIC‐luciferase system. (N) SW1353 cells treated with IL‐1β (10 ng/mL for 24 h) were cotransfected with the 8 × GTIIC‐luciferase plasmid, pRL‐TK, and each of 96 DUB expression plasmids. Firefly luciferase activity was normalised to Renilla luciferase activity; a normalised signal > 2.0‐fold that of the empty‐vector control was the prespecified gain‐of‐function hit threshold. The screen was repeated in three independent experiments. (O) Flow chart for the complementary loss‐of‐function DUB screen. (P) *GLI1* protein abundance after individual DUB depletion was quantified by densitometry relative to loading control and shcontrol; < 50% of shcontrol was the prespecified hit threshold. The corresponding immunoblots are shown in Figure S3A. This screen was repeated in three independent experiments. (Q, R) RT‐qPCR analysis of *BMI1*, *MYC*, and *BCL2* in SW1353 cells treated with IL‐1β (10 ng/mL for 24 h) after USP25 overexpression or shUSP25‐mediated depletion (also see Figure S3E–F). Data were obtained from three independent experiments with at least three biological replicates per condition and were analysed using unpaired two‐tailed Student's *t*‐tests. Significance levels were denoted as **p < .01; ***p < .001; ****p < .0001; ns (not significant). (S) Immunoblotting results showing levels of proteins associated with the Hedgehog pathway in USP25 or shUSP25 plasmid‐transfected SW1353 cells under IL‐1β‐induced OA‐like conditions. The data were representative of three independent experiments with similar results. Also refer to Figure S3G.

RT‐qPCR and immunoblotting showed higher *GLI1* expression in grossly degenerated than in paired relatively preserved cartilage from the same OA joints of 24 patients (Figure 1E and F). To examine whether *GLI1* tracked with the intensity of OA‐like chondrocyte injury, primary human chondrocytes were exposed to increasing concentrations of IL‐1β. *GLI1* mRNA increased in a concentration‐dependent manner, accompanied by progressive increases in the catabolic markers *MMP9* and *MMP13* and a corresponding decrease in the cartilage matrix marker *COL2A1* (Figure 1G–J). Immunoblotting confirmed the same concentration‐dependent changes at the protein level (Figure 1K). In parallel, CCK‐8 assays showed that IL‐1β progressively reduced cellular metabolic viability in primary human chondrocytes in a concentration‐ and time‐dependent manner (Figure 1L). Together, the independent cartilage datasets and paired human cartilage specimens supported an OA‐related tissue‐level expression pattern, while the primary chondrocyte experiments provided a complementary cellular context for subsequent mechanistic studies; the subchondral‐bone analysis served only as the initial candidate‐nomination step.

### 2.2 USP25 was prioritised as a *GLI1*‐regulating deubiquitinase

To identify DUBs that regulate *GLI1*, two complementary screens were conducted in SW1353 cells under IL‐1β‐induced OA‐like conditions (Figure 1M–P). In the gain‐of‐function screen, the prespecified hit threshold was a > 2.0‐fold increase in normalised 8×GTIIC GLI‐responsive luciferase activity relative to the empty‐vector control; eight DUBs met this criterion (Figure 1N). In the loss‐of‐function screen, the prespecified hit threshold was *GLI1* protein abundance < 50% of the shcontrol level after individual DUB depletion; four DUBs met this criterion (Figures 1O and P and S3A). Integration of the two screens prioritised USP25 for subsequent study. *USP25* mRNA expression correlated positively with the Hedgehog target genes *BMI1*, *BCL2*, and *MYC* in GSE51588 (Figure S3B–D), consistent with the RT‐qPCR results (Figure 1Q and R). Among the tested Hedgehog‐pathway components, USP25 overexpression or knockdown altered *GLI1* and its downstream targets without comparably changing the upstream regulators examined (Figure 1S). *GLI1* mRNA was not significantly altered by USP25 perturbation, whereas *GLI1* protein abundance changed markedly (Figure 1Q–S), supporting post‐translational regulation. Similar results were obtained in differentiated ATDC5 chondrocyte‐like cells (Figure S3E–G).

### 2.3 USP25 interacts with and stabilises *GLI1* in a deubiquitinase‐dependent manner

Immunofluorescence analysis was performed to examine the subcellular distribution of *GLI1* and USP25 in IL‐1β‐treated primary human chondrocytes, SW1353 cells, and differentiated ATDC5 cells. Representative images showed partial spatial overlap between USP25 and *GLI1* fluorescence signals, predominantly in the cytoplasmic compartment (Figures 2A and S4A). Quantitative image analysis using Pearson's correlation coefficient and thresholded Manders’ coefficients further supported partial fluorescence‐signal overlap between USP25 and *GLI1* across multiple cells, fields, and independent experiments. These imaging data are interpreted as descriptive spatial context, while reciprocal Co‐IP and GST pull‐down provide the principal evidence for biochemical association and direct binding, respectively. Reciprocal endogenous co‐immunoprecipitation in IL‐1β‐treated primary human chondrocytes detected an association between USP25 and *GLI1*; complementary co‐immunoprecipitation experiments in HEK293T, SW1353, and ATDC5 cells yielded consistent results (Figures 2B and S4B and C). GST pull‐down assays further supported direct binding between USP25 and *GLI1* (Figure 2C). Domain‐mapping experiments indicated that *GLI1* amino acids 235–387 (ZnF domain) interacted with the USP25 region spanning amino acids 169–657 (USP domain) (Figure 2D–G). The catalytically inactive USP25^C178A^ mutant did not increase *GLI1* protein abundance, supporting a requirement for USP25 deubiquitinase activity (Figure S4D). USP25 depletion reduced *GLI1* protein abundance in IL‐1β‐treated primary human chondrocytes and HEK293T cells, and MG132 restored *GLI1* levels, consistent with proteasome‐dependent regulation (Figures 2H and S4E). Cycloheximide‐chase experiments showed that wild‐type USP25 prolonged the *GLI1* protein half‐life, whereas USP25 silencing or catalytic inactivation accelerated *GLI1* loss (Figure 2I and J).

**FIGURE 2:**
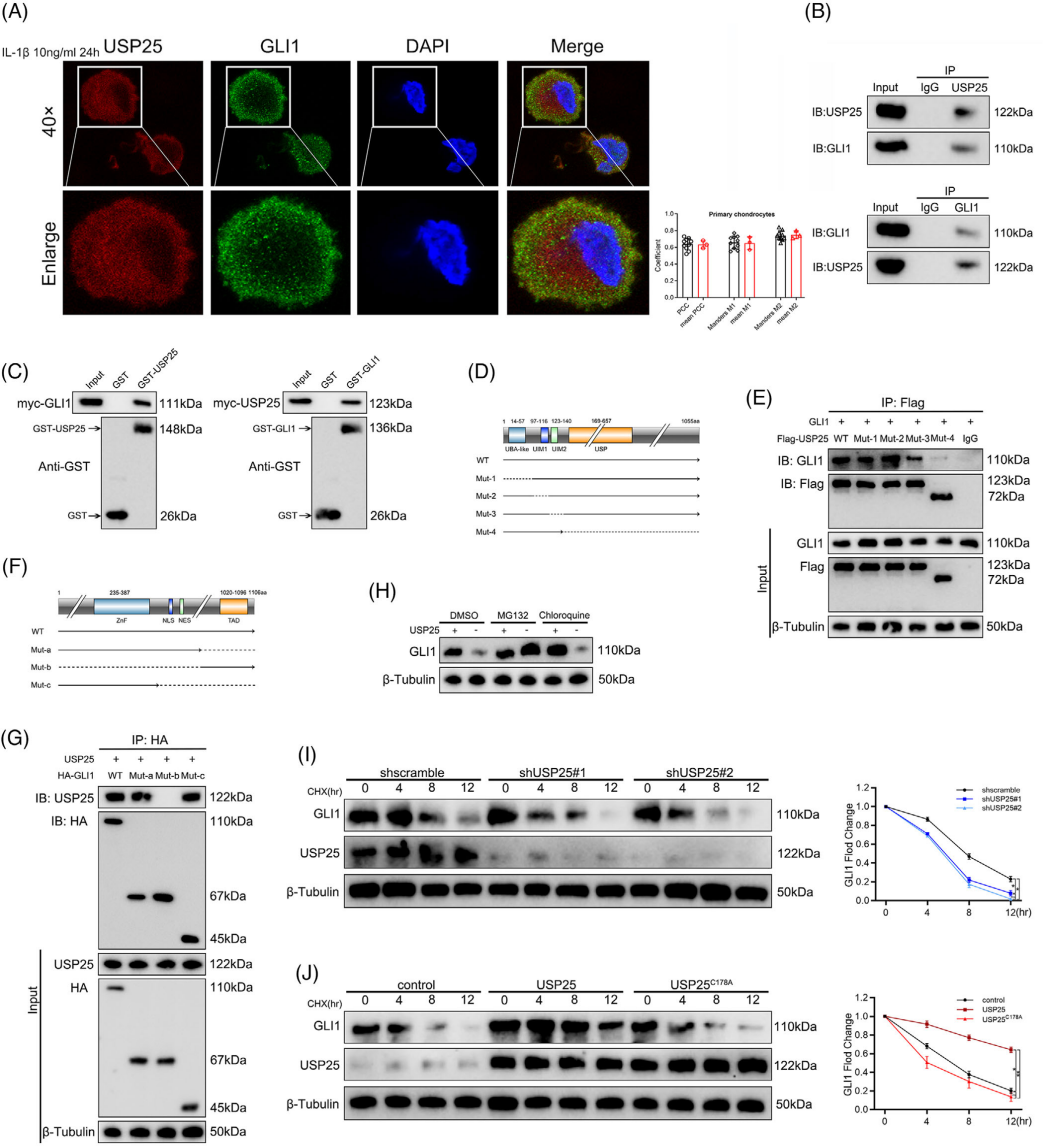
USP25 interacted with *GLI1* and stabilised its protein expression in a deubiquitinase activity‐dependent manner. (A) Representative immunofluorescence images showing the subcellular distributions and partial fluorescence‐signal overlap of USP25 (red) and *GLI1* (green) in primary human chondrocytes treated with IL‐1β (10 ng/mL) for 24 h. Nuclei were counterstained with DAPI (blue). Enlarged views of the indicated regions are shown below. Fluorescence‐signal overlap was quantified using Pearson's correlation coefficient (PCC) and thresholded Manders’ overlap coefficients (M1 and M2). For each independent experiment, 10 cells randomly selected from multiple non‐overlapping fields were analysed, and the mean value of the 10 cells was used as one experiment‐level value. Data are presented as mean ± SD from three independent experiments. Images from IL‐1β‐treated SW1353 cells and differentiated ATDC5 cells are shown in Figure S4A. (B) Reciprocal Co‐IP in IL‐1β‐treated primary human chondrocytes detected the endogenous USP25‐*GLI1* association. Complementary Co‐IP experiments in additional cell models are shown in Figure S4B and C. (C) GST pull‐down assay was performed to detect the interaction of USP25 and *GLI1*. (D–G) Schematic representation of the domain architecture of USP25 or *GLI1*. Full‐length and mutant plasmids of USP25 or *GLI1* were engineered and transfected into HEK293T cells. A Co‐IP test was conducted to investigate the binding domain of USP25 or *GLI1*. (H) IL‐1β‐treated primary human chondrocytes with USP25 depletion were treated with MG132 (10 µM) or chloroquine (50 µM) for 6 h, followed by immunoblotting of USP25 and *GLI1*. (I, J) shUSP25, USP25, or USP25^C178A^ plasmid‐transfected HEK293T cells were mixed with CHX (50 µM) for the indicated time intervals. Quantification was performed by densitometry. The experiments were performed independently three times with at least three biological replicates per condition. CHX time‐course data were analysed using two‐way ANOVA with multiplicity‐adjusted post hoc comparisons. Significance levels were denoted as *p < .05; **p < .01.

### 2.4 USP25 removed K48‐linked polyubiquitin chains from *GLI1*

Ubiquitination assays were performed to determine whether USP25 stabilises *GLI1* through deubiquitination. USP25 knockdown increased total *GLI1* ubiquitination in IL‐1β‐treated primary human chondrocytes and HEK293T cells, whereas wild‐type USP25 reduced *GLI1* ubiquitination; the catalytically inactive USP25C178A mutant did not produce the same effect (Figure 3A–H). In a cell‐free deubiquitination assay, purified wild‐type USP25, but not USP25^C178A^, reduced ubiquitination of purified *GLI1* (Figure 3I and J). Linkage‐specific assays in primary human chondrocytes and an extended ubiquitin‐linkage panel in HEK293T cells showed that USP25 preferentially reduced K48‐linked polyubiquitination of *GLI1* (Figure 3K and L). Together, these results support a model in which USP25 removes K48‐linked polyubiquitin chains from *GLI1* and limits its proteasomal degradation.

**FIGURE 3:**
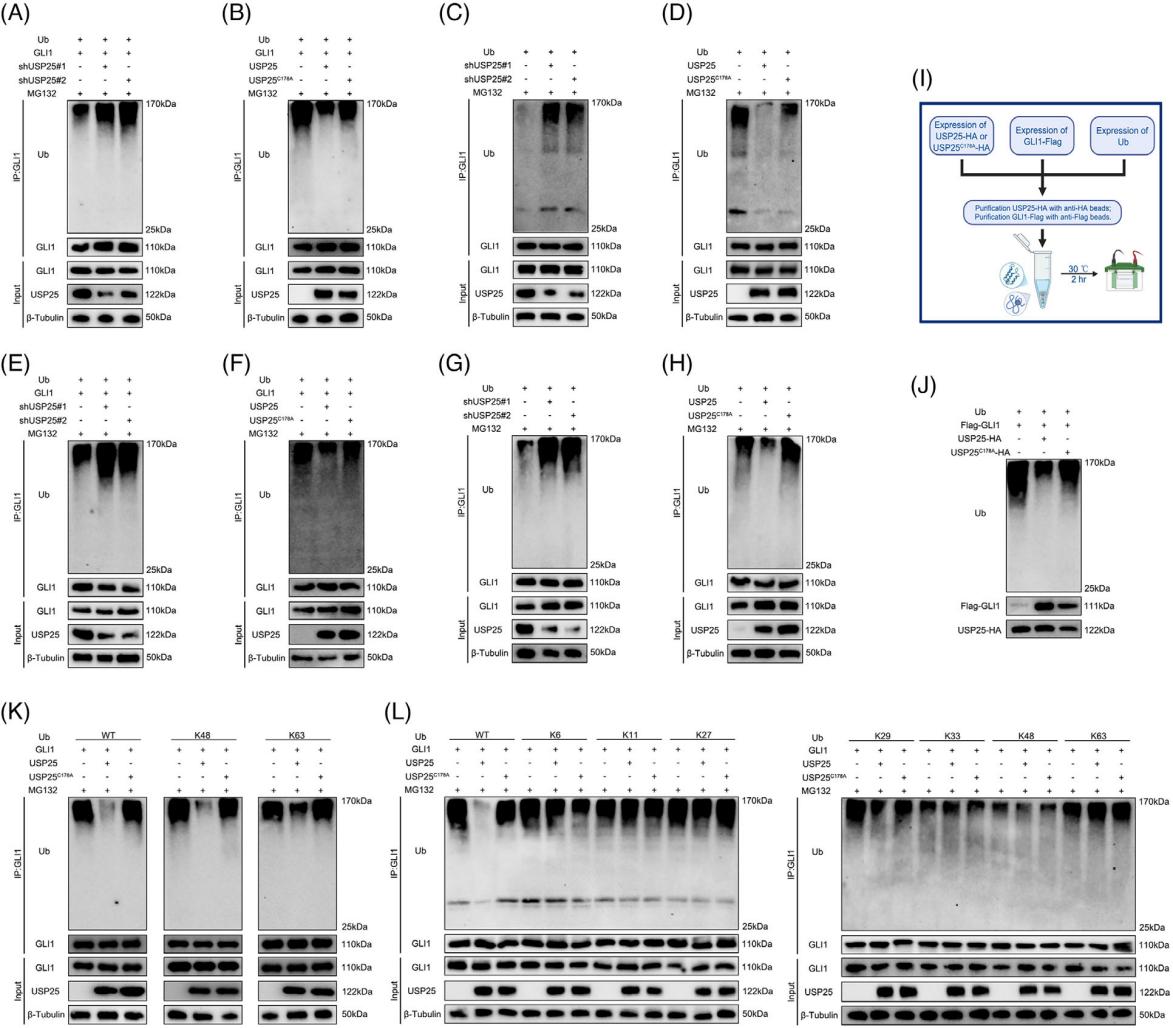
USP25 removed K48‐linked polyubiquitin moieties from *GLI1*. (A, B) The Ub assay was performed with exogenously overexpressed *GLI1*, Ub, and shUSP25/USP25/USP25^C178A^ in IL‐1β‐treated primary human chondrocytes treated with IL‐1β (10 ng/mL for 24 h). After 48 h, cells were treated with MG132 (10 µM), lysed, and immunoprecipitated. (C, D) IL‐1β‐treated primary human chondrocytes were cotransfected with Ub and shUSP25, treated with MG132 (10 µM), and immunoprecipitated to assess *GLI1* Ub. (E, F) The Ub assay was performed with exogenously overexpressed *GLI1*, Ub, and shUSP25/USP25/USP25^C178A^ in HEK293T cells. After 48 h, cells were treated with MG132 (10 µM), lysed, and immunoprecipitated. (G, H) HEK293T cells were cotransfected with Ub and shUSP25, treated with MG132 (10 µM), and immunoprecipitated to assess *GLI1* Ub. (I, J) In Ub assays, Ub and *GLI1*‐Flag plasmid‐transfected HEK293T cells were exposed to MG132 (10 µM) before purification with anti‐Flag magnetic beads, and incubation of USP25‐HA and USP25^C178A^‐HA with *GLI1*. (K) Ub assays of overexpressed *GLI1*, Ub (Ub/K48/K63) and USP25/USP25^C178A^ in IL‐1β‐treated primary human chondrocytes. Cells were treated with MG132 (10 µM) for 6 h after 48 h of transfection, and immunoprecipitation was performed to elucidate the Ub of *GLI1*. (L) Ub assays of overexpressed *GLI1*, Ub (Ub/K6/K11/K27/K29/K33/K48/K63) and USP25/USP25^C178A^ in HEK293T cells. Cells were treated with MG132 (10 µM) for 6 h after 48 h of transfection, and immunoprecipitation was performed to elucidate the Ub of *GLI1*. The biochemical deubiquitination assays were repeated independently three times with similar results. Representative blot images are shown above.

### 2.5 USP25 perturbation was associated with inflammatory and cartilage‐catabolic phenotypes in OA‐relevant models

To assess the functional consequences of USP25 perturbation, the catabolic markers MMP9 and MMP13, the cartilage‐matrix marker COL2A1, cellular metabolic viability, and DNA synthesis were evaluated. USP25 knockdown decreased MMP9 and MMP13 and increased COL2A1 at the mRNA and protein levels in IL‐1β‐treated SW1353 and differentiated ATDC5 cells (Figure S5A–C and G). USP25 overexpression produced the opposite pattern, whereas the catalytically inactive USP25^C178A^ mutant showed little or no activity (Figures S5D–F and S5H). CCK‐8 assays indicated increased cellular metabolic viability after USP25 depletion, whereas EdU incorporation indicated increased DNA synthesis in the SW1353 and ATDC5 cell‐line experiments. USP25 overexpression produced the opposite effects, whereas USP25^C178A^ again showed reduced activity (Figure S5I–N). These findings support a deubiquitinase‐activity‐dependent association between USP25 and inflammatory or cartilage‐catabolic phenotypes in the cell models examined.

Furthermore, to assess USP25 perturbation in vivo, we used both intra‐articular shUSP25 delivery in C57BL/6J (WT) mice and an inducible *Usp25^fl/fl^*; *Col2a1*‐Cre^ERT^+ conditional line (Figures S6 and S7A). In the conditional line, corn‐oil‐treated, non‐induced *Usp25^fl/fl^*; *Col2a1*‐Cre^ERT^+ mice were designated *Usp25*‐ctrl, whereas littermates from the same conditional line received tamoxifen (30 mg/kg/day for 5 consecutive days) to induce *Col2a1*‐Cre^ERT^‐mediated *Usp25* recombination and were designated *Usp25*‐cKO. *Usp25* mRNA and USP25 protein abundance in articular cartilage were lower in the tamoxifen‐induced *Usp25*‐cKO arm than in WT mice receiving intra‐articular shUSP25 under both sham and DMM conditions (Figure 4A and B), and IHC of whole‐joint sections showed reduced USP25 staining in the articular‐cartilage compartment (Figure 4C and D). These data indicate greater tissue‐level USP25 depletion in the inducible conditional arm than with intra‐articular shUSP25 delivery under the tested conditions. H&E and Safranin O staining, OARSI grading, osteophyte scoring, and IHC were then compared between the corn‐oil‐treated *Usp25*‐ctrl arm and the tamoxifen‐induced *Usp25*‐cKO arm under matched sham or DMM surgical conditions (Figures 4E–N, S6, and S7B). Compared with the corn‐oil‐treated *Usp25*‐ctrl arm, tamoxifen‐induced *Col2a1*‐Cre^ERT^‐mediated *Usp25* depletion was associated with reduced DMM‐related cartilage pathology and corresponding changes in MMP9, MMP13, and COL2A1 staining under the reported induction conditions. Because tamoxifen exposure and Usp25 recombination were linked in this comparison, the in vivo result is interpreted as an association under the reported induction conditions, with the effects of the induction regimen and recombination not separable in this design. Furthermore, immunoblotting showed higher USP25 protein abundance in grossly degenerated than in paired relatively preserved cartilage from the same OA joints of 24 patients (Figure 4O). Spearman's analysis showed a modest positive correlation between USP25 and *GLI1* protein abundance (*r* = 0.43, *p* = .037; Figure 4P), supporting coordinated expression at the tissue level.

**FIGURE 4:**
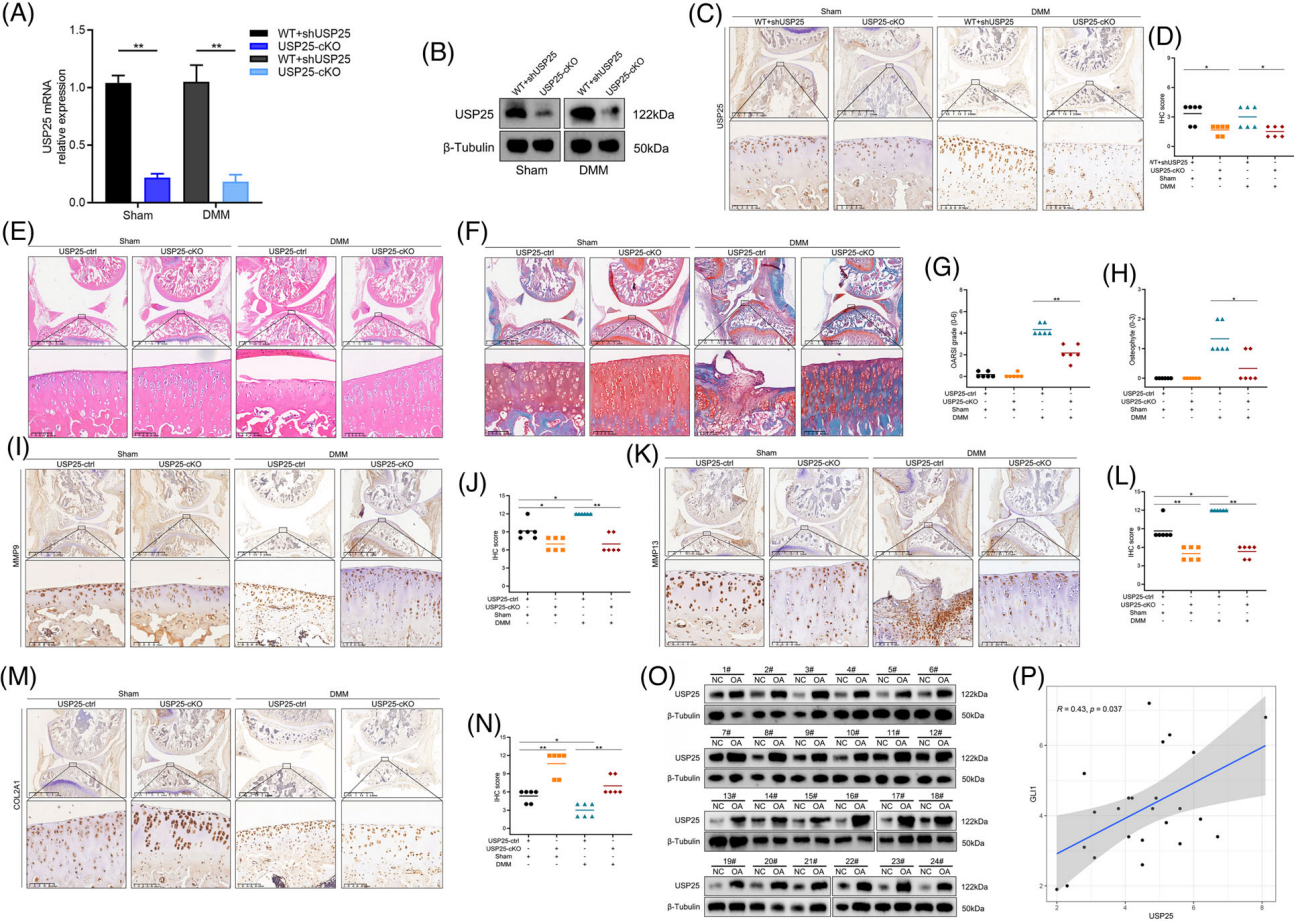
Association of USP25 perturbation with OA‐related molecular and cartilage phenotypes. (A, B) RT–qPCR analysis of *Usp25* mRNA (A) and immunoblotting of USP25 protein (B) analyses of *Usp25* expression in articular cartilage from WT mice receiving an intra‐articular injection of 10 µL shUSP25 adenovirus (1 × 10^9 PFU; WT + shUSP25) and tamoxifen‐induced *Usp25*‐cKO mice subjected to sham or DMM surgery (Groups 1 vs. 7 and Groups 2 vs. 8, respectively), *n* = 6 mice/group. β‐Tubulin was used as the loading control. Significance levels were denoted as **p < .01. (C, D) IHC staining and quantification of USP25 in the same comparison, *n* = 6 mice/group. (E–H) H&E staining (E), Safranin O staining (F), and OARSI grade (G) and osteophyte score (H) comparing corn‐oil‐treated *Usp25*‐ctrl mice with tamoxifen‐induced *Usp25*‐cKO mice under matched sham or DMM surgery (Groups 3 vs. 7 and Groups 4 vs. 8), *n* = 6 mice/group. (I–N) IHC staining and semiquantitative scoring of MMP9, MMP13, and COL2A1 expression and related readouts in the same animal cohorts, *n* = 6 mice/group. The individual mouse was the biological and statistical unit. Continuous RT‐qPCR and densitometric immunoblot measurements in a and b were analysed using unpaired two‐tailed Student's *t*‐tests; semiquantitative IHC, OARSI grade, and osteophyte score comparisons in c–n were analysed using the Mann–Whitney *U* test for the prespecified two‐group contrasts within each surgical condition. Representative histological images were selected from the predefined medial weight‐bearing region after blinded scoring. Significance levels were denoted as *p < .05; **p < .01. (O) Immunoblotting of USP25 in paired grossly degenerated and relatively preserved cartilage from the same OA joints of 24 patients. (P) Spearman's correlation between densitometrically quantified USP25 and *GLI1* protein abundance in the analysed human cartilage samples (*r* = 0.43, *p* = .037), indicating a modest positive tissue‐level association.

### 2.6 *GLI1* substantially, but incompletely, mediated USP25‐associated phenotypes

We next assessed the extent to which *GLI1* mediates the functional effects of USP25. In IL‐1β‐treated SW1353 cells and differentiated ATDC5 cells, *GLI1* overexpression partially reversed the molecular and CCK‐8‐derived cellular metabolic viability changes associated with USP25 knockdown (Figure 5A–C). In DMM mice, intra‐articular *GLI1* overexpression partially reversed the reduction in MMP9 staining observed in the tamoxifen‐induced *Usp25*‐cKO arm relative to the corn‐oil‐treated *Usp25*‐ctrl arm (Figures 5D and E, S6, and S7C). Our findings showed that *GLI1* overexpression partially restored MMP9 expression in the DMM model, supporting an in vivo contribution of *GLI1* to the molecular response associated with USP25 perturbation. However, studies incorporating structural cartilage outcomes and refined conditional models are needed to further verify the extent of *GLI1* involvement in OA progression. To extend the cellular rescue analysis to a primary human model, bidirectional rescue experiments were performed in IL‐1β‐treated primary human chondrocytes. Consistent with the findings in IL‐1β‐treated SW1353 cells and differentiated ATDC5 cells, USP25 knockdown reduced MMP9 protein expression, whereas simultaneous *GLI1* overexpression partially restored MMP9 expression. Conversely, USP25 overexpression increased MMP9 abundance, while knockdown of *GLI1* using either of two independent shRNAs markedly attenuated, but did not fully abolish, this effect (Figure 5F). CCK‐8 analysis further showed that *GLI1* overexpression partially reversed the increase in cellular metabolic viability associated with USP25 knockdown in primary human chondrocytes (Figure 5G). In contrast, depletion of *GLI1* with either sh*GLI1*#1 or sh*GLI1*#2 attenuated, but did not fully eliminate, the decrease in cellular metabolic viability associated with USP25 overexpression, with comparable effects observed for the two independent shRNAs (Figure 5H). Collectively, the bidirectional rescue experiments in established cell lines and primary human chondrocytes provide cellular epistasis evidence that *GLI1* mediates a substantial, but not exclusive, component of the inflammatory and cartilage‐catabolic phenotypes associated with USP25 perturbation. The incomplete rescue in both directions is consistent with contributions from additional USP25 substrates or signalling pathways. The in vivo experiment provides complementary molecular evidence for *GLI1* involvement, based on MMP9 IHC.

**FIGURE 5:**
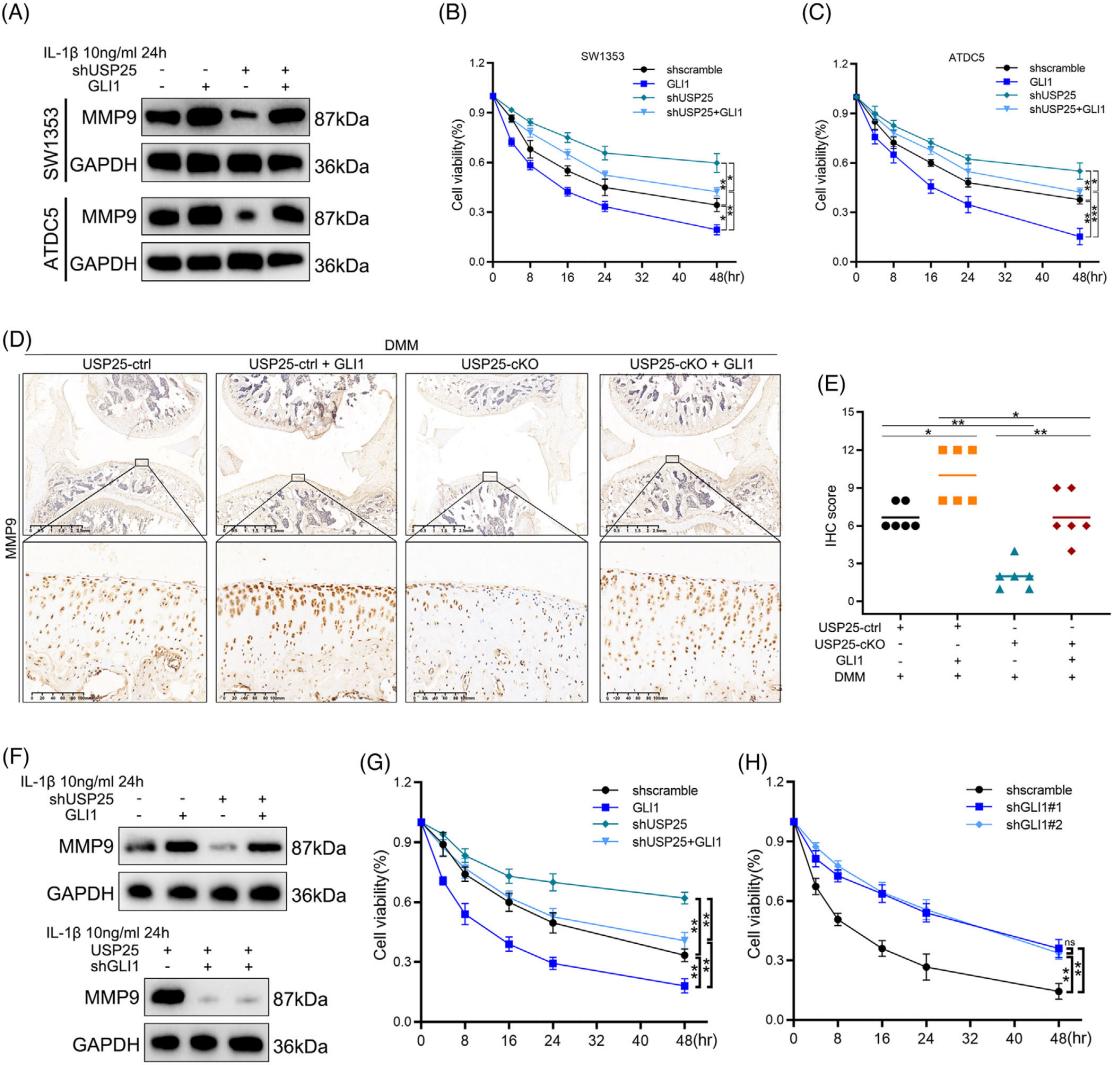
*GLI1* substantially, but incompletely, mediated USP25‐associated phenotypes. (A) Immunoblotting confirmed the efficiency of USP25 knockdown and *GLI1* overexpression in SW1353 cells and differentiated ATDC5 cells treated with IL‐1β (10 ng/mL for 24 h). (B, C) CCK‐8 assessment of cellular metabolic viability after USP25 knockdown with or without *GLI1* overexpression in the same cell models. Cell‐based experiments were performed independently three times with at least three biological replicates per condition; time‐course data were analysed using two‐way ANOVA with multiplicity‐adjusted post hoc comparisons. (D, E) MMP9 IHC and semiquantitative scoring in DMM‐operated Groups 4, 5, 8, and 9, *n* = 6 mice/group. The individual mouse was the biological and statistical unit. Comparisons across the four animal groups were analysed using the Kruskal–Wallis test followed by Dunn's multiple‐comparison test, with Group 8 vs. Group 9 prespecified as the principal *GLI1*‐rescue contrast. (F) Immunoblotting of MMP9 in primary human chondrocytes treated with IL‐1β (10 ng/mL for 24 h) after USP25 knockdown with or without *GLI1* overexpression and after USP25 overexpression with either shScramble or one of two independent *GLI1*‐targeting shRNAs. GAPDH was used as the loading control. (G, H) CCK‐8 assessment of cellular metabolic viability in the corresponding primary‐human‐chondrocyte rescue experiments. Experiments were performed independently three times with at least three biological replicates per condition; treatment and time were analysed jointly using two‐way ANOVA with multiplicity‐adjusted post hoc comparisons. Significance levels were denoted as *p < .05; **p < .01; ns, not significant.

### 2.7 *GLI1* positively modulates USP25 transcription

The GTRD database (https://gtrd20‐06.biouml.org) revealed that USP25 is a potential transcriptional target gene of *GLI1*. Consistently, *GLI1* silencing reduced endogenous USP25 protein abundance and the corresponding *USP25/Usp25* transcript levels in IL‐1β‐treated SW1353 cells and differentiated ATDC5 cells, whereas ectopic USP25 expression restored USP25 abundance in *GLI1*‐depleted cells (Figure 6A–E). HOCOMOCO v13 analysis identified three candidate *GLI1*‐binding regions within the tested *USP25* promoter fragment (Figure 6F and G). To determine whether *GLI1*‐dependent transcription was mediated by a specific predicted site, the three sites were mutated individually or in combination, and the resulting promoter constructs were compared side by side with the wild‐type reporter. *GLI1* overexpression markedly increased wild‐type *USP25* promoter activity. Mutation of R1 or R2 alone did not abolish *GLI1* responsiveness, and the R2/R1 double‐mutant construct, in which R3 remained intact, also retained significant *GLI1*‐induced activity. In contrast, the R3‐mutant reporter and all combined mutant constructs containing the R3 mutation failed to show significant *GLI1*‐dependent promoter activation (Figure 6H). ChIP‐qPCR further demonstrated significant *GLI1* enrichment at the R3‐containing promoter region, but not at the R1‐ or R2‐containing regions, in both IL‐1β‐treated SW1353 cells and differentiated ATDC5 cells (Figure 6I). Together, the site‐directed promoter mutagenesis and ChIP‐qPCR data identify R3 as the principal functional *GLI1*‐responsive site within the tested *USP25* promoter region. Through this site, *GLI1* acts as an upstream transcriptional regulator that reinforces USP25 expression within the reciprocal regulatory circuit.

**FIGURE 6:**
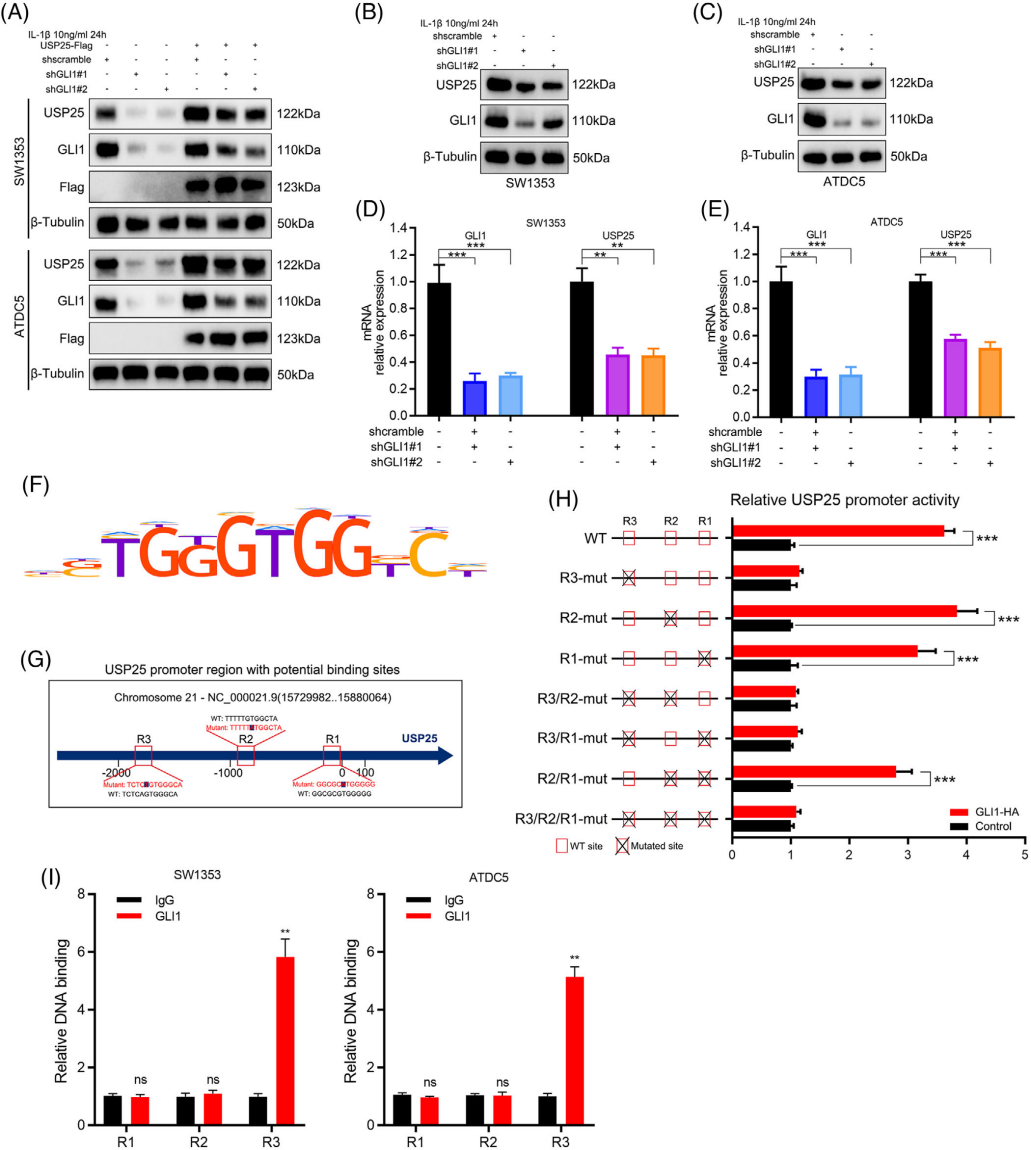
*GLI1* positively regulated USP25 expression at the transcriptional level. (A) Western blotting was used to assess the protein levels of *GLI1* and USP25 in SW1353 cells and differentiated ATDC5 cells treated with IL‐1β (10 ng/mL for 24 h) following USP25 overexpression and *GLI1* silencing. (B, C) After *GLI1* silencing in the same IL‐1β‐treated cell models, Western blotting was used to assess changes in *GLI1* and USP25 protein expression, while (D, E) RT‐qPCR was used to assess *GLI1* and *USP25* mRNA levels. (F) The *USP25* promoter region was screened and predicted for potential *GLI1* binding sites in the HOCOMOCO database. (G) A schematic diagram of the *USP25* promoter region with potential *GLI1* binding sites. Wild‐type and mutant *GLI1*‐binding sequences are shown. R1 (Region 1), R2 (Region 2), and R3 (Region 3) indicate three sites in the *USP25* promoter containing the potential binding sites. (H) Side‐by‐side dual‐luciferase reporter analysis of the wild‐type promoter and the explicitly labelled R3‐mut, R2‐mut, R1‐mut, R3/R2‐mut, R3/R1‐mut, R2/R1‐mut, and R3/R2/R1‐mut constructs in the presence of *GLI1*‐HA or control vector. Renilla luciferase activity served as the internal reference. The reporter assays were performed independently three times with three biological replicates per condition. Data are presented as mean ± SD and were analysed using unpaired two‐tailed Student's *t*‐tests for the indicated comparisons. Significance levels were denoted as ***p < .001. (I) ChIP‐qPCR analysis of *GLI1* enrichment at the R1‐, R2‐, and R3‐containing *USP25* promoter regions in SW1353 cells and differentiated ATDC5 cells treated with IL‐1β (10 ng/mL for 24 h). Data are presented as mean ± SD from three independent experiments. Prespecified pairwise comparisons were analysed using unpaired two‐tailed Student's *t*‐tests. Significance levels were denoted as **p < .01.

### 2.8 Chondroprotective activity and exploratory target‐dependence analysis of AZ1

The preclinical pharmacological activity of AZ1, a reported dual inhibitor of USP25 and USP28, was evaluated in OA‐relevant models. At the cellular level, AZ1 reduced MMP9 and MMP13 protein levels, increased COL2A1 expression, and increased CCK‐8‐derived cellular metabolic viability in IL‐1β‐treated SW1353 cells and differentiated ATDC5 cells (Figure 7A–C). In the DMM mouse model, intra‐articular AZ1 treatment was associated with reduced histological cartilage damage, OARSI grade, and osteophyte score and produced corresponding changes in MMP9, MMP13, and COL2A1 staining (Figures 7D–M, S6A, and S7D). Together, these findings demonstrate preclinical pharmacological activity of AZ1 in the tested cellular and DMM models.

**FIGURE 7:**
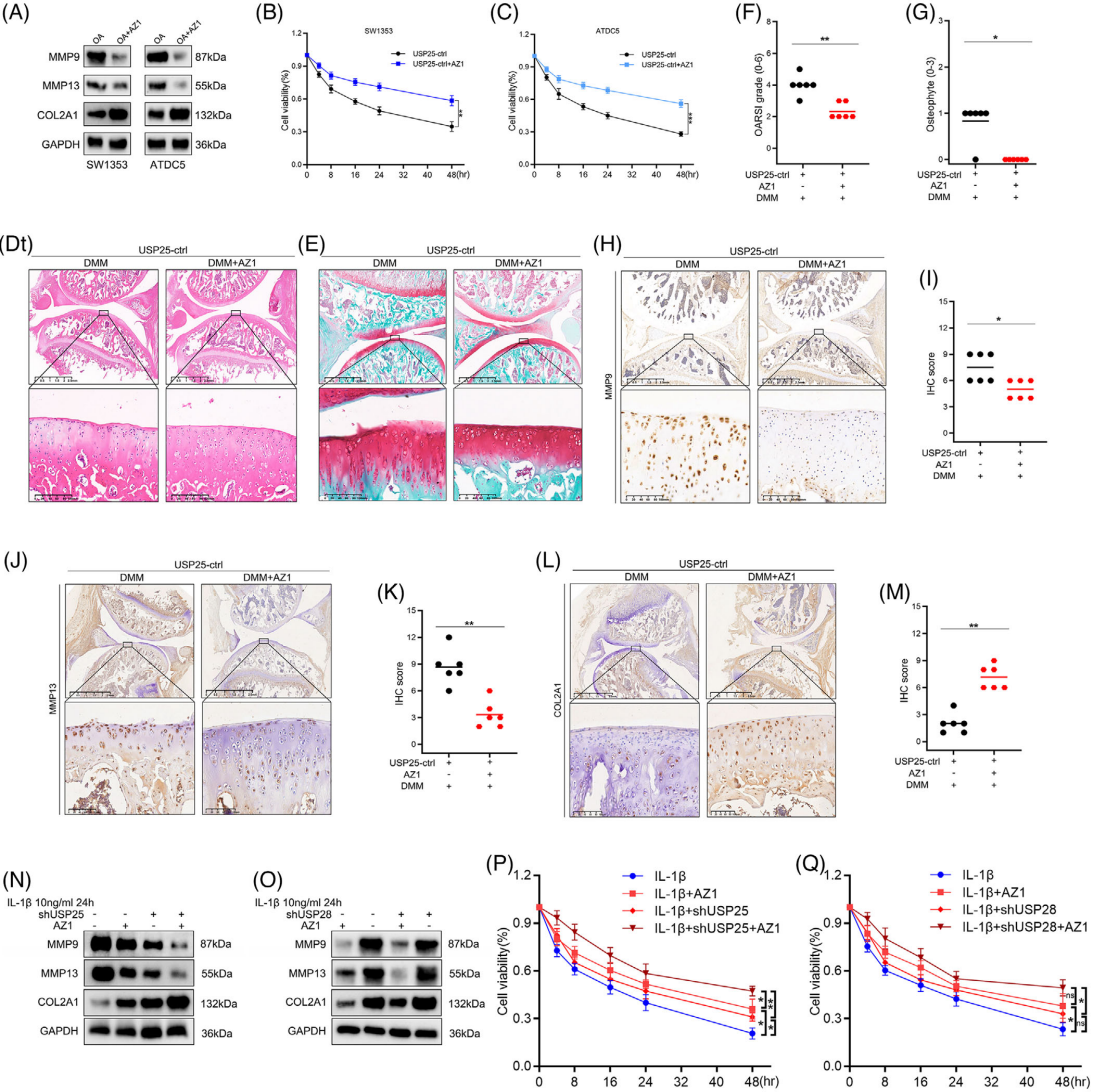
Preclinical pharmacological activity and exploratory target‐dependence analysis of AZ1. (A) Immunoblotting of MMP9, MMP13, and COL2A1 in SW1353 cells and differentiated ATDC5 cells exposed to IL‐1β (10 ng/mL for 24 h) and treated with vehicle or AZ1 (10 µM for 24 h). (B, C) CCK‐8 assessment of cellular metabolic activity/viability in the same cell models; treatment and time were analysed jointly using two‐way ANOVA with multiplicity‐adjusted post hoc comparisons. (D–M) Histological and immunohistochemical analyses of the prespecified DMM‐operated *Usp25*‐ctrl comparison (Group 4 vs. Group 6), *n* = 6 mice/group; Group 6 received intra‐articular AZ1 (10 µL at 0.422 mg/mL once weekly for 2 consecutive weeks). The individual mouse was the biological and statistical unit. OARSI grades, osteophyte scores, and **semiquantitative** IHC scores were analysed using the Mann–Whitney *U* test for the prespecified Group 4 vs. Group 6 comparison. (N, O) Immunoblotting of MMP9, MMP13, and COL2A1 in primary human chondrocytes exposed to IL‐1β (10 ng/mL for 24 h), transduced with shcontrol, shUSP25 (N), or shUSP28 (O), and treated with or without AZ1 (10 µM for 24 h). Representative immunoblots from three independent experiments are shown. (P, Q) CCK‐8 assessment of cellular metabolic viability in the corresponding USP25‐ or USP28‐knockdown experiments at the indicated time points. These experiments were performed independently three times with at least three biological replicates per condition; treatment and time were analysed jointly using two‐way ANOVA with multiplicity‐adjusted post hoc comparisons. Significance levels were denoted as *p < .05; **p < .01; ns, not significant.

To further explore the target dependence of AZ1, IL‐1β‐treated primary human chondrocytes were subjected to USP25 or USP28 knockdown and then treated with or without AZ1. USP25 knockdown alone decreased MMP9 and MMP13 expression, increased COL2A1 expression, and improved cellular metabolic viability, thereby reproducing a substantial portion of the AZ1‐associated phenotype. Addition of AZ1 to USP25‐silenced cells produced a further change in the same protective direction, although the additional reduction in MMP9 was relatively modest (Figure 7N). A residual AZ1‐associated increase in cellular metabolic viability was also observed after USP25 knockdown (Figure 7P). Thus, USP25 depletion substantially phenocopied AZ1 treatment, and the smaller residual response after USP25 depletion supports partial USP25 dependence with additional target contributions remaining possible. By contrast, USP28 knockdown alone did not reproduce the AZ1‐associated molecular and metabolic phenotype. AZ1 reduced MMP9 and MMP13 and increased COL2A1 in both the presence and absence of USP28 knockdown (Figure 7O), and its effect on cellular metabolic viability was similarly retained after USP28 depletion (Figure 7Q). These findings suggest that USP28 is not the major mediator of the AZ1‐associated phenotype under the conditions tested. Residual activity is compatible with contributions from additional molecular targets or pathways. Because AZ1 was not tested in *Usp25*‐cKO mice, its degree of USP25 dependence in vivo remains unresolved. Collectively, the data support AZ1 as a proof‐of‐concept pharmacological probe with chondroprotective activity and partial USP25 dependence. A summary flowchart is provided in the Supplementary Graphical Abstract.

## 3 DISCUSSION

Osteoarthritis is a joint disorder involving the degeneration of articular cartilage.^1^ Several studies have investigated the cellular functions in the tissue microenvironment of OA patients and have identified novel bio‐indices and targeted therapeutic regimens.^29^, ^30^ However, effective disease‐modifying targets remain limited. In the present study, GSE51588 served only as a hypothesis‐generating subchondral‐bone dataset that nominated *GLI1*, whereas independent cartilage datasets, paired human cartilage specimens, and primary chondrocyte experiments provided the tissue‐ and cell‐relevant support for the mechanistic work that followed. Across public bulk datasets, *GLI1* was increased in OA cartilage, decreased in OA synovium, and unchanged in peripheral blood mononuclear cells, indicating compartment‐dependent rather than uniform whole‐joint or systemic dysregulation. Because these analyses were performed on bulk tissues, they cannot determine whether the between‐compartment differences reflect cell‐intrinsic regulation, differences in cellular composition, or both, nor can they identify the joint‐resident population primarily responsible for the in vivo phenotype. The concentration‐dependent increase in *GLI1* observed after IL‐1β exposure in our primary human chondrocytes should likewise be interpreted as a response within the specific OA‐like inflammatory conditions used here rather than as a universal OA response. Consistent with such context dependence, Thompson et al. reported that recombinant IHH activated Hedgehog signalling without increasing MMP13, ADAMTS5, or matrix loss in human and bovine cartilage models, and that IL‐1β attenuated IHH‐induced Hedgehog signalling.^31^ A related example is the Wnt/β‐catenin–PTHrP negative‐feedback loop, which maintains cartilage homeostasis in primary chondrocytes but becomes disrupted in pathological chondrocyte states, further illustrating the context dependence of signalling networks in OA.^32^ Taken together, these observations indicate that Hedgehog/*GLI1* biology varies with species, tissue state, mode of pathway activation, and inflammatory or injury context. These observations provide a disease‐relevant context for the *GLI1*‐centred mechanistic studies that followed. Accordingly, the principal advance of the present study is the identification of *GLI1* as a USP25‐associated substrate, the demonstration that USP25 removes K48‐linked polyubiquitin chains to stabilise *GLI1*, and evidence that *GLI1* reciprocally reinforces USP25 transcription. In the paired human cartilage cohort, grossly degenerated and relatively preserved regions from the same OA joints were compared, and the modest positive USP25‐*GLI1* correlation (Spearman's *r* = 0.43) supports coordinated tissue‐level expression. Recent human multi‐omics and spatial studies emphasise the heterogeneity of chondrocyte states and their zonal organisation in OA cartilage. Accordingly, cell‐type and zonal localisation of the human USP25‐*GLI1* relationship remain important directions for dedicated tissue IHC/IF and single‐cell or spatial analyses.

A previous OA study linked USP25 to NLRP3 inflammasome activation by showing that USP25 deubiquitinates and stabilises thioredoxin‐interacting protein (TXNIP), thereby aggravating IL‐1β‐induced chondrocyte injury and OA progression.^22^ The present findings are consistent with the reported pro‐inflammatory role of USP25 but extend it to a distinct substrate and signalling context. Specifically, we identify *GLI1* as a direct USP25‐associated substrate and show that removal of K48‐linked polyubiquitin chains protects *GLI1* from proteasomal degradation. The USP25‐*GLI1* circuit should therefore be interpreted as one mechanistic branch of USP25 biology in the tested OA‐relevant models, rather than the sole or complete mechanism by which USP25 acts in OA. The USP25‐*GLI1* and previously reported USP25‐TXNIP‐NLRP3 pathways may coexist, but their functional hierarchy and interaction remain unresolved. Because NLRP3 activation, caspase‐1 processing, and mature IL‐1β release were not directly assessed in the present study, we cannot determine whether these two branches operate upstream of, downstream of, or in parallel with one another. The comparison with other OA‐associated DUBs further illustrates the substrate dependence of deubiquitination. USP14, like USP25, promotes cartilage‐catabolic responses by enhancing NF‐κB signalling through IκBα degradation.^24^ In contrast, USP7, USP3, and USP49 protect chondrocytes by suppressing endoplasmic reticulum stress/NF‐κB signalling, TRAF6‐dependent inflammation, or Wnt/β‐catenin signalling, respectively.^23^, ^25^, ^26^ USP15 and UCH37 also exert chondroprotective effects by maintaining ERK2‐ or ALK5‐dependent TGF‐β signalling and cartilage matrix synthesis.^27^, ^28^ Thus, DUB activity is not uniformly protective or pathogenic in OA; its biological effect depends on the substrate, ubiquitin linkage, and signalling pathway involved. Our results place USP25 among the pro‐catabolic DUBs and identify *GLI1* stabilisation as an additional mechanism through which USP25 may disrupt cartilage homeostasis. The reciprocal rescue experiments further refine the pathway interpretation. In SW1353 and ATDC5 cells and in IL‐1β‐treated primary chondrocytes, *GLI1* gain‐ or loss‐of‐function substantially countered the effects of USP25 perturbation, but the rescue was incomplete in both directions. These cellular epistasis data therefore support *GLI1* as a substantial, but not exclusive, downstream mediator of USP25‐associated inflammatory and cartilage‐catabolic phenotypes, consistent with contributions from additional USP25 substrates or signalling pathways. By contrast, the in vivo rescue evidence was based on the partial restoration of MMP9 IHC by *GLI1* overexpression in the DMM model. These findings support the involvement of *GLI1* in the molecular response in vivo. However, further evaluation incorporating structural histology, OARSI grading, and osteophyte assessment is needed to more comprehensively characterise and substantiate the contribution of *GLI1* to the structural DMM phenotype.

*GLI1* is a transcription factor and therefore lacks readily druggable pockets, making direct small‐molecule inhibition challenging.^33^, ^34^ Accordingly, targeting regulatory proteins that control *GLI1* stability may represent a more feasible strategy. Recent advances have increasingly highlighted DUBs as pharmacologically tractable regulators of transcription factors,^35^ and several small‐molecule DUB inhibitors have been investigated in different diseases.^36^, ^37^ The two complementary functional screens prioritised USP25 for mechanistic study as a regulator of *GLI1* protein stability and ubiquitination. Mechanistically, USP25 stabilised *GLI1* by removing K48‐linked polyubiquitin chains, and this regulatory branch was associated with inflammatory and cartilage‐catabolic phenotypes in the tested OA‐relevant models. In turn, *GLI1* activated USP25 transcription predominantly through the R3 promoter site, thereby reinforcing USP25 expression within the reciprocal regulatory circuit. Together, these data identify USP25 as a positive regulator of *GLI1* stability and activity and define a *GLI1*‐centred mechanistic branch of USP25 biology that warrants further investigation. In vivo, the inducible conditional experiment showed an association between tamoxifen‐induced *Col2a1*‐CreER‐mediated Usp25 depletion and reduced DMM‐related cartilage pathology under the reported induction conditions. Because the control arm was not tamoxifen matched, this design cannot isolate the effect of Usp25 recombination from the induction regimen. Consistently, AZ1 showed preclinical pharmacological activity in primary chondrocytes and mouse OA models. The target‐dependence experiments in primary human chondrocytes further supported partial USP25 dependence of the AZ1‐associated phenotype. Nevertheless, the current findings do not establish the expression patterns of USP25 and *GLI1* across all joint‐resident cell populations or demonstrate direct associations with radiographic grade, histological severity, pain intensity, or functional impairment. Further validation in larger, clinically annotated human cohorts is therefore required before definitive translational conclusions can be drawn.

Studies have indicated that USP25 modulates substrate protein levels via the ubiquitin‐proteasome pathway, which depends on the recognition of specific ubiquitin chain positions or linkage types by its catalytic structural domains.^38^ Here, it was observed that USP25 binds and modulates *GLI1* via post‐translational modification, which may suggest that USP25 targets various other substrates with similar ubiquitin chain types in a relatively non‐specific manner. Analogous to this mechanism, USP25 can enhance the stability of the substrate protein, hence inhibiting its degradation via the proteasome pathway. Research indicates that USP25 stabilises SERCA2a (sarcoplasmic reticulum calcium ATPase) through de‐Ub, thus preserving calcium handling capacity and mitigating pathological cardiac hypertrophy.^37^ Moreover, USP25 can deubiquitinate FOXO3 to inhibit Ang II‐induced vascular remodelling and may have potential in treating diseases involving excessive vascular remodelling.^39^ Furthermore, USP25 regulates the stability of signalling molecules and is involved in inflammatory and immunological responses. It can modulate the TLR4‐mediated inflammatory response and engage in immunological responses through K48‐linked ubiquitin chains derived from TRAF3.^40^ Research has demonstrated that USP25 is expressed in cardiac and skeletal muscle and mediates muscle differentiation. The USP25 isoform is increased during myogenesis and interacts with three sarcomeric proteins, contributing to muscle differentiation and maintenance, which are implicated in the etiology of severe myopathies.^41^ In addition, it is abnormally high in various tumour‐related diseases, including leukaemia, colorectal cancer, and lung cancer, making it a potential therapeutic target.^42^, ^43^, ^44^ Therefore, it is imperative to further investigate the mechanistic details of USP25.

*GLI1* is a central transcriptional effector of Hedgehog signalling, and altered *GLI1*‐associated transcriptional networks have previously been implicated in human OA cartilage. *GLI1* abundance and activity are controlled by several post‐translational modifications, including ubiquitination. Previous studies, conducted predominantly in tumour models, have identified multiple DUBs that regulate *GLI1* or Hedgehog signalling. USP5 directly stabilises *GLI1* in osteosarcoma, USP21 regulates *GLI1* stability and transcriptional activity at the centrosome, and USP48 is induced by *GLI1* and in turn stabilises *GLI1* in glioblastoma.^45^, ^46^, ^47^ USP37 and USP39 also enhance Hedgehog/*GLI1* signalling through broader regulatory mechanisms in breast and lung cancer models.^48^, ^49^ These reports establish deubiquitination as an important mechanism controlling *GLI1*, but they do not define how *GLI1* stability is maintained in OA cartilage. The present study extends this concept to OA by identifying USP25 as a *GLI1* deubiquitinase, defining K48‐linked polyubiquitin chains as the relevant linkage, and validating the functional relationship in primary chondrocytes and mouse OA models. Moreover, *GLI1* bound the *USP25* promoter and increased *USP25* transcription. This reciprocal regulation resembles the *GLI1*‐USP48 relationship described in glioblastoma,^46^ but identifies a different DUB and establishes its relevance to inflammatory and cartilage‐catabolic changes in OA. Accordingly, the principal advance of the present study is not the general concept that DUBs can stabilise *GLI1*, but the identification and mechanistic characterisation of a USP25‐*GLI1* regulatory circuit in OA.

Since the discovery of USP25 in 1999,^50^ USP28 was rediscovered in 2001 using sequence alignment.^51^ The levels of USP28 and USP25 are increased in several cancers, neurological disorders, and immune system diseases.^52^, ^53^, ^54^ Furthermore, USP25 is significantly expressed in musculoskeletal disorders; however, aberrant USP28 expression is not linked to these conditions.^55^ Silencing or inhibiting either USP28 or USP25 can significantly mitigate the advancement of these disorders, indicating that they may serve as promising novel therapeutic targets.^42^, ^43^, ^44^, ^45^, ^46^, ^47^, ^48^, ^49^, ^50^, ^51^, ^52^, ^53^, ^54^, ^55^ However, the substantial similarity between USP25 and USP28 results in poor selectivity of inhibitors. Structurally, USP25 and USP28 share 48% and 76% sequence homology and similarity, respectively. Their catalytic USP domains indicate 57% sequence homology, including similar insertion sites, about 170 amino acids long.^55^ Among the AZ‐series compounds, the inhibitory impacts of AZ1 to AZ4 decline progressively, with AZ1 exhibiting the most potent inhibitory impact. The AZ series has effective binding and inhibitory properties for both USP25 and USP28.^56^ Intra‐articular administration was selected to increase local joint exposure, but local delivery does not establish molecular selectivity or eliminate off‐target activity. In primary chondrocytes, USP25 knockdown reproduced a substantial portion of the AZ1‐associated protective phenotype, while a smaller residual response to AZ1 remained after USP25 depletion, supporting partial rather than exclusive USP25 dependence. In contrast, USP28 knockdown neither reproduced the protective phenotype nor abolished the response to AZ1, suggesting that USP28 was not the major mediator under the conditions tested. The current data therefore support partial USP25 dependence but do not justify describing AZ1 as a USP25‐specific inhibitor. Experiments using AZ1 in *Usp25*‐cKO mice, rescue with knockdown‐resistant USP25, direct target‐engagement assays, and chemically distinct selective inhibitors will be required to define in vivo target dependence and additional targets. These findings provide preclinical proof‐of‐concept.

The findings support a bidirectional USP25‐*GLI1* regulatory circuit centred on *GLI1* that is associated with inflammatory and cartilage‐catabolic changes in OA models, while identifying AZ1 as a pharmacological probe with potential chondroprotective activity. Several limitations should nevertheless be emphasised. First, GSE51588 profiles subchondral bone and therefore serves only as a hypothesis‐generating discovery dataset; it should be viewed as a candidate‐nomination step. Although independent cartilage transcriptomic datasets, paired human cartilage specimens, and primary‐chondrocyte experiments substantially strengthened the tissue relevance of the findings, they do not eliminate the initial tissue mismatch in the discovery step. In addition, although the paired human cartilage cohort included 24 patients, complete Kellgren–Lawrence grades, OARSI scores, cartilage degeneration scores, pain scores, and functional assessments were not available for all specimens. Therefore, formal correlations between USP25/*GLI1* expression and clinical OA severity could not be performed. The IL‐1β dose‐gradient experiment models graded OA‐like inflammatory injury in our primary chondrocyte system, but it does not establish that *GLI1* responds similarly across all OA contexts or substitute for clinical severity analysis in well‐annotated patient cohorts. Second, the study did not include IHC or IF localisation of USP25 and *GLI1* in human OA tissue, nor single‐cell or spatial transcriptomic analyses. Because the public transcriptomic datasets are bulk tissue profiles, the observed between‐compartment differences may reflect both cell‐intrinsic regulation and variation in cellular composition. The IF findings in primary chondrocytes and the IHC findings in mouse cartilage support chondrocyte relevance, but they cannot determine whether USP25 and *GLI1* are preferentially expressed in human chondrocytes, synovial cells, subchondral bone cells, or other joint‐resident populations. Third, in vitro and animal models do not fully reproduce the heterogeneity of human OA. *Col2a1*‐CreER is not strictly specific to adult articular chondrocytes and may induce recombination in other *Col2a1*‐lineage cells within the joint. In addition, the central conditional‐model comparison was not tamoxifen‐matched: the *Usp25*‐ctrl arm consisted of corn‐oil‐treated, non‐induced *Usp25^fl/fl^*; *Col2a1*‐Cre^ERT^+ mice, whereas the *Usp25*‐cKO arm received tamoxifen to induce recombination, and no tamoxifen‐treated non‐recombined control was included. Therefore, the effects of the induction regimen cannot be completely separated from those of *Usp25* recombination. Although RT‐qPCR and immunoblotting of articular cartilage and IHC of whole‐joint sections confirmed substantial USP25 reduction in the cartilage compartment, Cre‐reporter lineage tracing and tissue‐resolved genomic recombination analyses were not performed. Consequently, the observed association between the tamoxifen‐induced *Usp25*‐cKO arm and reduced DMM‐related cartilage pathology cannot be attributed exclusively to *Usp25* depletion in adult articular chondrocytes or solely to *Usp25* recombination. It may partly reflect recombination in other Col2a1‐lineage joint cells and/or a contribution of the induction regimen. Future studies should include tamoxifen‐matched Cre‐negative or otherwise non‐recombined controls, together with Cre‐reporter lineage tracing, cell‐ or tissue‐resolved recombination analysis, and a more adult‐articular‐chondrocyte‐restricted genetic model to define the responsible effect more precisely. Fourth, AZ1 is a reported dual USP25/USP28 inhibitor, and the present target‐dependence experiments indicate partial USP25 dependence of the AZ1‐associated phenotype. Future studies using more selective compounds, direct target‐engagement assays, pharmacokinetic analyses, and longer‐term safety assessment will help define joint‐level selectivity and translational potential.

## 4 CONCLUSIONS

In summary, our data identify a *GLI1*‐centred mechanistic branch in which USP25 removes K48‐linked polyubiquitin chains from *GLI1* and limits its proteasomal degradation, while *GLI1* reinforces USP25 transcription. This reciprocal circuit is associated with inflammatory and cartilage‐catabolic phenotypes in the tested OA‐relevant models. The paired human cartilage findings provide tissue‐level support for coordinated USP25‐*GLI1* expression within OA joints, while further clinically annotated and spatially resolved studies will help define its human relevance. AZ1 showed preclinical pharmacological activity in the tested cell and DMM models and is interpreted here as a proof‐of‐concept pharmacological probe with partial USP25 dependence. Further studies are warranted to define target selectivity, long‐term safety, and translational relevance.

## 5 METHODS

### 5.1 Data collection and preprocessing

Gene‐expression datasets were obtained from the GEO database. GSE51588, comprising 40 OA and 10 normal subchondral bone samples before quality control, was used as the hypothesis‐generating discovery dataset. Following log2 transformation, normalisation, probe annotation, principal component analysis, and quality control, 35 OA and 6 normal samples were retained for downstream analyses. Independent cartilage datasets (GSE178557, GSE114007, and GSE129147), synovial tissue datasets (GSE1919, GSE12021, GSE55235, and GSE55457), and the peripheral blood mononuclear cell dataset GSE300310 were analysed to evaluate compartment‐specific *GLI1* expression across OA‐related tissues. Detailed information on the datasets and platforms is provided in Table S1.

Datasets from the same tissue type were preprocessed separately and then integrated. Batch effects were corrected using the ComBat algorithm in the sva R package, and the effectiveness of correction was assessed by dimensionality‐reduction analysis before and after correction. The resulting tissue‐specific expression matrices were used for differential expression, conventional GSEA, and *GLI1*‐based single‐gene enrichment analyses.

### 5.2 WGCNA

This assay was carried out using the R WGCNA package to screen co‐expressed genes and modules. Briefly, each gene's median absolute deviation (MAD) values were assessed, and the top 8000 genes with the highest MAD values were selected. Then, an undirected network was established based on the absolute correlations between genes to construct co‐expression networks. The deepSplit parameter was set to 2. A soft threshold of 20 yielded a scale‐free topology model fit of 0.9, which promoted the detection of co‐expression modules. Subsequently, an adjacency matrix was derived from the expression matrix, which in turn was translated into a topological matrix. Clustering of genes was performed using the topological overlap measure and the average‐linkage hierarchical clustering approach. Modules were detected using the dynamic tree cut algorithm and similar modules were merged at cut height 0.25. Based on the standard of the hybrid dynamic shearing tree approach, a minimum of 50 genes per network module was selected. Next, eigengenes for each module were determined, and a cluster analysis was carried out. Modules in proximity were combined with the detectCutHeight parameter specified at 0.25 to complete the modules. Genes that did not align with any single module were classified into the grey module. The interrelations among genes within each module were assessed using Pearson correlation coefficients. Lastly, hub genes modulating the promotion or inhibition of each OA module were identified based on an MM score of >0.8 and the GS score of >0.5.

### 5.3 Identification of *GLI1*

Differential gene expression analyses were conducted with the R limma package. DEGs were identified based on a |log_2_(FC)| > 1 and an adjusted *p*‐value < .05. Using the DEGs as the core dataset, predicted PPI relationships were obtained from the STRING database, and a confidence threshold > 0.4 was set to screen for reliable interactions. An undirected PPI network was constructed with proteins as nodes and interaction relationships as edges. Hub proteins were screened by setting the topological parameter Degree ≥ 10, and module clustering was combined to explore functional interaction modules.

### 5.4 GSEA

The Hedgehog pathway‐related data were retrieved from the KEGG database. Based on the information provided in the datasets, the samples were categorised into normal control and OA groups. Subsequently, all genes’ mRNA levels were systematically analysed via R software. Furthermore, a single‐gene enrichment method was employed to perform enrichment analysis in OA via R software.

### 5.5 Cell culture

Primary human articular chondrocytes, human chondrosarcoma cells (SW1353), undifferentiated murine mesenchymal cells (ATDC5), and human embryonic kidney cells (HEK293T) were employed for in vitro experiments. ATDC5 cells were differentiated with insulin, transferrin, sodium selenite, and vitamin C for 2–3 weeks. Primary human chondrocytes, SW1353 cells, and differentiated ATDC5 chondrocyte‐like cells were treated with IL‐1β (10 ng/mL for 24 h) to establish OA‐like inflammatory conditions. Primary human chondrocytes were used for graded disease‐relevance experiments (0, 5, 10, or 20 ng/mL IL‐1β), endogenous interaction and protein‐stability analyses, ubiquitination assays, bidirectional USP25/*GLI1* rescue experiments, and AZ1 target‐dependence analyses. IL‐1β‐treated SW1353 cells and differentiated ATDC5 cells were used as complementary chondrocyte‐like models, whereas HEK293T cells were used for experiments requiring high transfection efficiency or detailed biochemical mapping. Cells were maintained in RPMI 1640 or DMEM supplemented with 10% fetal bovine serum and 100 U/mL penicillin/streptomycin at 37°C in 5% CO_2_ and were routinely tested for mycoplasma contamination.

AZ1, a reported dual USP25/USP28 inhibitor (Cayman Chemical, Item No. 40335), was prepared in 0.1% DMSO in sterile PBS according to the experimental record. For cellular experiments, AZ1 was applied at a final concentration of 10 µM for 24 h, with the corresponding vehicle used for matched controls.^57^ For target‐dependence experiments, primary human chondrocytes transduced with shcontrol, shUSP25, or shUSP28 were exposed to IL‐1β (10 ng/mL for 24 h) and treated with or without AZ1 under the same AZ1 treatment conditions. For in vivo experiments, 10 µL AZ1 at 0.422 mg/mL in 0.1% DMSO in sterile PBS was administered intra‐articularly once weekly for 2 consecutive weeks beginning at week 7. The dosing scheme was selected with reference to prior OA pharmacology using AZ1^22^ and was applied consistently across the corresponding experiments.

### 5.6 In vivo experiments

C57BL/6J mice were procured from Shenzhen Lingfu Top Biotechnology Company. The inducible conditional line was generated by breeding *Usp25^fl/fl^* mice with *Col2a1*‐Cre^ERT^ transgenic mice, yielding *Usp25^fl/fl^*; *Col2a1*‐Cre^ERT^+ animals. The designation *Usp25*‐ctrl refers to mice from this conditional line that received corn‐oil vehicle and therefore remained non‐induced; *Usp25*‐cKO refers to mice from the same *Usp25^fl/fl^*; *Col2a1*‐Cre^ERT^+ line that received intraperitoneal tamoxifen (30 mg/kg/day, CAS 10540‐29‐1, Sigma) for 5 consecutive days to induce Cre^ERT^‐mediated *Usp25* recombination. The *Usp25^fl/fl^*; *Col2a1*‐Cre^ERT^+ conditional line was provided by Shanghai Model Organisms Center.

Equal numbers of male and female mice were included at a 1:1 ratio, and the mice were 8–12 weeks old at the start of the experiment. For each in vivo experiment, eligible mice were assigned to experimental groups using a computer‐generated block‐randomisation sequence after confirmation of eligibility. Group size was *n* = 6 mice per group. No formal a priori sample‐size calculation was performed. The group size of six mice was selected based on preliminary experiments, previously published DMM studies, and the principle of using the minimum number of animals required to obtain reproducible histological and molecular outcomes. Prespecified exclusion criteria included perioperative death, postoperative joint infection, fracture or other major surgical complications, technically unsuccessful DMM surgery, and tissue‐processing artefacts that prevented reliable histological assessment. No animals were excluded after randomisation. The experimental procedure and the nine experimental groups are detailed in Figure S6A. Corn oil or tamoxifen was administered at week 0; sham or DMM surgery was performed at week 2; treadmill exercise began at week 3 and continued for 4 weeks; the indicated intra‐articular interventions began at week 7 and were administered once weekly for 2 consecutive weeks; mice were euthanised at week 9. The nine groups were defined as follows: Groups 1–2, WT mice receiving intra‐articular shUSP25 under sham or DMM conditions; Groups 3–6, corn‐oil‐treated *Usp25*‐ctrl mice subjected to sham or DMM, with control adenovirus, *GLI1* adenovirus, or AZ1 as indicated; and Groups 7–9, tamoxifen‐induced *Usp25*‐cKO mice subjected to sham or DMM, with control adenovirus or *GLI1* adenovirus as indicated. All mice underwent the same exercise protocol. DMM surgery combined with treadmill exercise was used to establish the OA model. The right knee joint capsule was incised, followed by transection of the medial meniscotibial ligament. The ligament was not transected after joint opening in the sham group. One week after surgery, mice underwent treadmill exercise at 20 m/min with a 0° incline for 30 min per day over 4 consecutive weeks. For different experimental groups, 10 µL of shUSP25 adenoviruses [1 × 10^9^ plaque‐forming units (PFUs)], shcontrol adenoviruses, *GLI1* adenoviruses (1 × 10^9^ PFUs), or 10 µL of AZ1 [0.422 mg/mL] were administered into the mice's right knee joint once a week for 2 consecutive weeks.

The prespecified analytical contrasts are detailed in Figure S7A–D. Treatment and group codes were concealed from investigators performing section selection, histological scoring, IHC quantification, and data analysis until the prespecified analyses were completed. All animals were kept in the Laboratory Animal Department at Sun Yat‐sen University. All in vivo assays were authorised by the Experimental Animal Care Commission of Sun Yat‐sen University and followed the ethical standards established by the institutional ethics board and agreed with the NC3Rs ARRIVE requirements.

### 5.7 Patient samples

Paired articular cartilage specimens were obtained from 24 patients through the Seventh Affiliated Hospital of Sun Yat‐sen University and Shanghai Outdo Biotech Company. For each patient, one region with pronounced macroscopic degeneration and one relatively preserved region from the same OA joint were selected according to gross tissue appearance and analysed as a matched pair. The paired comparator was the relatively preserved region from the same OA joint; clinically non‐OA donor cartilage was not included. Exact zone‐matched anatomical annotation was not available for all specimens; pairing was therefore defined by within‐joint macroscopic tissue status. The patient‐level information used in the present analysis comprised within‐patient specimen pairing and macroscopic tissue status; complete Kellgren–Lawrence grades, OARSI scores, cartilage degeneration scores, pain scores, functional assessments, and other clinical annotations were not available for all specimens. Accordingly, the human analyses focused on paired tissue‐level expression differences and the association between USP25 and *GLI1* protein abundance. The protocol was approved by the Medical Ethics Committee of the Seventh Affiliated Hospital of Sun Yat‐sen University, and informed consent was obtained from all participants.

### 5.8 Western blotting and DUB screening

Proteins were extracted using RIPA buffer, separated by SDS–PAGE, and transferred onto PVDF membranes. After blocking, the membranes were incubated with primary antibodies overnight at 4°C and HRP‐conjugated secondary antibodies for 1 h. Signals were detected using ECL in automatic exposure mode, and only unsaturated images were used for densitometric analysis. Uncropped membranes, molecular‐weight markers, lane annotations, and loading controls are provided in the raw‐data files.

For the gain‐of‐function screen, SW1353 cells under IL‐1β‐induced OA‐like conditions were cotransfected with the 8 × GTIIC reporter, pRL‐TK, and each of the 96 DUB expression plasmids. Firefly luciferase activity was normalised to Renilla luciferase activity, and a normalised signal > 2.0‐fold that of the empty‐vector control was prespecified as a screening hit. For the complementary loss‐of‐function screen, each DUB was individually depleted in SW1353 cells, GLI1 protein abundance was quantified densitometrically relative to the corresponding loading control and shcontrol, and a normalised GLI1 level < 50% of shcontrol was prespecified as a hit. Each screening stage was repeated in three independent experiments. The two hit sets were compared, and candidates supported by both screening directions were prioritised for mechanistic follow‐up.

### 5.9 Luciferase reporter assay

The 8× GTIIC‐luciferase reporter vector was used to quantify GLI1‐mediated transcriptional activity. To examine GLI1‐dependent regulation of the *USP25* promoter, a genomic fragment spanning –2000 to +100 bp relative to the transcription start site was synthesised (Lingfu Top Biotechnology, Shenzhen, China) and cloned into the promoterless pGL3‐Basic vector. Candidate GLI1‐binding motifs were predicted using the HOCOMOCO v13 database (https://hocomoco13.autosome.org/) and designated R1, R2, and R3. Inverse PCR‐based site‐directed mutagenesis was used to generate the wild‐type reporter and seven mutant reporters: R3‐mut, R2‐mut, R1‐mut, R3/R2‐mut, R3/R1‐mut, R2/R1‐mut, and R3/R2/R1‐mut. HEK293T cells were cotransfected with each *USP25* promoter reporter, either GLI1‐HA or the corresponding control vector, and pRL‐TK encoding Renilla luciferase as the normalisation control. After 48 h, firefly and Renilla luciferase activities were measured using a dual‐luciferase reporter kit (Vazyme), and relative promoter activity was calculated by normalising firefly luciferase activity to Renilla luciferase activity.

### 5.10 Co‐immunoprecipitation

Following plasmid transfection or treatments for 24 h, cells were harvested, lysed with IP buffer [1 mL comprising NaCl (150 mM), Tris‐HCl (20 mM; pH 7.4), EDTA (1 mM), and 1% NP‐ 40]. The IP buffer was augmented with PhosSTOP (Ref: 001,04,837,906, Roche, USA) and protease inhibitors (Ref: 001,04,132,693, Roche). The lysate was then ultrasonicated and centrifuged (12 000 rpm, 15 min, 4°C). Of the supernatant, 50 µL was retained, while the remainder was mixed (overnight, 4°C) with Protein A/G Magnetic Beads (B23202, Biomake, USA) and antibody on a rotator. Subsequently, the beads underwent four rinses with cold IP buffer, followed by boiling in loading buffer for 15 min, and centrifugation. The supernatant was employed for immunoblotting analysis. The antibodies used in the study were listed in Table S2.

### 5.11 GST pull‐down assays

PCR was employed to amplify the full‐length USP25 and GLI1 genes using plasmid templates. The amplified genes were then inserted into the pGEX‐4T‐1 vector to construct recombinant plasmids, which were transformed into *Escherichia coli* BL21, followed by protein induction using 100 µM IPTG at 16°C for 12 h. Subsequently, the bacterial pellets were resuspended in RIPA lysis buffer, disrupted using ultrasonication, and centrifuged to collect supernatants, which were treated with glutathione‐agarose resin for 2 h on a rotary shaker at 4°C. Bound proteins were eluted by boiling the beads in SDS loading buffer and were assessed by immunoblotting.

### 5.12 Ubiquitination assays

The pRK5‐HA‐ubiquitin plasmids were acquired from the Abcam website. The ubiquitin coding sequence was then PCR‐amplified and inserted into the vector to generate recombinant constructs. Cell Ub analysis was carried out by cotransfecting primary human chondrocytes or HEK293T cells with GLI1 and Ub expression plasmids, either alone or in combination with USP25^C178A^ or USP25 for 36 h. Then, to inhibit proteasomal degradation, cells were treated with MG132 (10 µM) for 6 h. Cell lysates were treated with SDS lysis buffer [50 µL comprising Tris‐HCl (20 mM, pH 7.4), NaCl (150 mM), EDTA (1 mM), 1% SDS], heated for 10 min at 99°C to denature proteins, and diluted with IP buffer (500 µL). The subsequent immunoprecipitation steps adhered to conventional Co‐IP methods. Transfection of the HEK293T cells was carried out with Ub and Flag‐GLI1 plasmids for the in vitro Ub experiment. After 48 h, cells were exposed to 10 µM MG132 for 6 h, and Flag‐tagged GLI1 was isolated using anti‐Flag magnetic beads. Subsequently, purified ubiquitinated GLI1‐Flag was incubated with purified USP25‐HA and USP25^C178A^‐HA in DUB reaction buffer (20 mM Tris‐Cl, pH 7.5, 5 mM MgCl_2_, 2 mM DTT, 10 mM ATP) at 37°C for 2 h.

### 5.13 Total RNA isolation and RT‐qPCR

Whole RNA of treated cells was harvested using TRIzol (Vazyme), and then employed for cDNA synthesis, per the protocol of the HiScript® III RT SuperMix for qPCR (Vazyme). RT‐qPCR was carried out with ChamQ Universal SYBR qPCR Master Mix (Vazyme), and ACTB was set as an endogenous reference. Bio‐Rad CFX Manager 2.1 was used for quantification. The primers were listed in Table S3.

### 5.14 Plasmid construction, transfection, and viral infection

From human cellular cDNA, the USP25 and GLI1 truncated and full‐length sequences were acquired via PCR, and then cloned into the pHAGE‐HA or pHAGE‐Flag vector. The USP25^C178A^ construct was generated from the USP25 plasmid by site‐directed mutagenesis using inverse PCR. Sangon Biotech designed and synthesised the gene‐specific small hairpin RNA sequences, which were then incorporated into the pLKO.1 vector. Table S4 lists the shRNA targeting sequences. Polyethyleneimine (25 kDa, Polysciences, USA) was used for transient transfection. Cotransfection of cells was carried out with the pMD2.G envelope, target gene, and psPAX packaging plasmids for 72 h. After filtering the supernatant (0.45 µm filter), differentiated ATDC5 cells and SW1353 cells were transduced using viral supernatant (1 mL) and polybrene (1 µL; 40804ES76, Yeasen, Shanghai, China) for 8 h. Subsequently, 1 µg/mL puromycin dihydrochloride (ST551, Beyotime, China) was employed to systematically screen the transduced cells for 2 weeks. The indicated gene was then evaluated for its overexpression or knockdown efficiency via RT‐qPCR and immunoblotting.

### 5.15 CCK‐8 assay

Cellular metabolic viability was assessed using the Cell Counting Kit‐8 (CCK‐8, RiboBio, Guangzhou, China) according to the manufacturer's instructions. Briefly, chondrocytes were seeded into 96‐well plates at a density of 2000 cells per well in 100 µL of complete culture medium. After incubation for 24, 48, and 72 h at 37°C in a 5% CO_2_ humidified atmosphere, 10 µL of CCK‐8 solution was added to each well. The plates were then incubated for an additional 2 h at 37°C. The absorbance of each well was measured at a wavelength of 450 nm using a microplate reader (Thermo Fisher Scientific, Waltham, MA, USA). For experiments assessed across multiple time points, treatment and time were analysed jointly by two‐way ANOVA followed by Tukey's post hoc test.

### 5.16 EdU incorporation assay

DNA synthesis was assessed using the 5‐ethynyl‐2'‐deoxyuridine (EdU) incorporation assay (RiboBio, Guangzhou, China). The EdU assay was used for the SW1353 and ATDC5 cell‐line experiments. Chondrocytes were seeded into 96‐well plates at a density of 2000 cells per well and cultured until reaching approximately 50% confluence. The cells were then incubated with culture medium containing 20 µM EdU for 2 h at 37°C. Following incubation, the cells were fixed with 4% paraformaldehyde for 30 min at room temperature, washed three times with PBS, and permeabilised with 0.5% Triton X‐100 for 10 min. After another three washes with PBS, the cells were stained with Hoechst 33342 for 30 min to label the nuclei. EdU‐positive cells were visualised and counted under a fluorescence microscope (Leica Microsystems, Wetzlar, Germany). The EdU incorporation index was calculated as the percentage of EdU‐positive cells relative to the total number of Hoechst‐stained cells and was interpreted specifically as a measure of DNA synthesis.

### 5.17 Haematoxylin‐eosin staining (H&E)

For histological assessment, serial sagittal knee sections (5 µm) through the medial compartment were reviewed using consistent anatomical landmarks. Sections at comparable levels showing the medial femoral condyle and medial tibial plateau were selected before group identities were revealed. The predefined region of interest for quantitative histological assessment was the central weight‐bearing articular cartilage of the medial tibial plateau. Selected sections were deparaffinised and rehydrated, stained with haematoxylin for 5 min, differentiated in 1% acid ethanol, counterstained with eosin for 5 min, dehydrated, mounted, and examined by bright‐field microscopy. OARSI grade (0–6) and osteophyte score (0–3) were independently reassessed by two investigators blinded to group allocation using the same predefined weight‐bearing region. Each mouse was treated as one biological and statistical unit, and the mean of the two observers' scores was used for analysis. The best available representative images from the predefined region of interest were selected only after completion of the blinded scoring.

### 5.18 Safranin‐O‐Fast Green staining

Cartilage and bone tissues were analysed via the Safranin‐O‐Fast Green staining technique. First, the samples were deparaffinised, dehydrated, and stained with Fast Green dye for an optimised time to highlight the bone tissue. Then, the cartilage was stained by submerging it in Safranin‐O dye. Extra dye was removed with an acidic differentiation solution. Lastly, the sections were dehydrated, mounted, and comprehensively evaluated within the predefined medial weight‐bearing region.

### 5.19 Immunohistochemical staining

For IHC, paraffin‐embedded tissue sections were incubated with antibodies against USP25, MMP9, MMP13, or COL2A1 antibodies for 1 h at 37°C, followed by a HRP‐labelled streptavidin for 10 min. Subsequently, the sections were stained with haematoxylin and DAB. IHC images and scores were obtained from the predefined central weight‐bearing region of the medial tibial plateau used for histological assessment. The stained tissue area was assessed using a scoring system: 1 for 1%–25% stained area, 2 for 26%–50%, 3 for 51%–75%, and 4 for 76%–100%. The staining intensity was assessed on a scale from 0 (no coloration) to 3 (bright yellow, brown, and dark brown). The final IHC score was calculated by multiplying the area percentage score by the staining intensity score. Three observers blinded to group allocation performed the assessment. Scores from multiple images or observers were aggregated to one mouse‐level value; individual fields and sections were not treated as independent replicates.

### 5.20 Immunofluorescence (IF) staining

Primary human chondrocytes, SW1353 cells, and differentiated ATDC5 chondrocyte‐like cells were cultured on glass coverslips in 6‐well plates and transfected with the indicated USP25 and/or GLI1 constructs for 48 h. Cells were fixed with 4% paraformaldehyde for 15 min, permeabilised with 0.1% Triton X‐100 for 10 min, incubated with primary antibodies overnight at 4°C, and then probed with fluorescent secondary antibodies for 1 h at room temperature. Nuclei were counterstained, and fluorescence microscopy was used to assess the subcellular distributions of USP25 and GLI1 and their descriptive spatial compatibility. Images were acquired from multiple randomly selected, non‐overlapping fields across independent experiments using identical acquisition settings. Quantitative fluorescence‐overlap analysis was performed using Fiji/ImageJ. After background subtraction, individual cells were defined as regions of interest. For each analysed condition in each independent experiment, 10 cells were randomly selected from multiple non‐overlapping fields. Pearson's correlation coefficient (PCC) and thresholded Manders’ overlap coefficients (M1 and M2) were calculated for each cell using identical analysis criteria across all images. PCC quantified the pixel‐wise fluorescence‐intensity correlation between the USP25 and GLI1 channels; M1 represented the fraction of total USP25 fluorescence intensity located in GLI1‐positive pixels, and M2 represented the fraction of total GLI1 fluorescence intensity located in USP25‐positive pixels. The values obtained from the 10 cells were averaged to generate one experiment‐level PCC, M1, and M2 value. The analysis was independently repeated three times, and the three experiment‐level values were summarised as mean ± SD. These metrics were used as descriptive measures of spatial fluorescence overlap and were not interpreted as evidence of molecular interaction.

### 5.21 ChIP‐qPCR

ChIP‐qPCR assays were performed using the SimpleChIP® Enzymatic Chromatin IP Kit (#9003, CST, Danvers, MA, USA) according to the manufacturer's instructions with minor modifications. Cells transfected with indicated plasmids were cross‐linked with 1% formaldehyde for 10 min at room temperature, quenched with 0.125 M glycine (#7005, CST) for 5 min. Cells were harvested, washed with ice‐cold PBS, lysed for nuclei isolation, and chromatin was digested to 200–500 bp fragments with micrococcal nuclease. An aliquot (10%) of soluble chromatin was saved as input control. The remaining chromatin was incubated with indicated antibodies or normal rabbit IgG (negative control) overnight at 4°C with rotation, followed by incubation with Protein G magnetic beads for 2 h at 4°C. Beads were collected with a magnetic stand and washed sequentially with low‐salt, high‐salt, LiCl, and TE buffers. Immune complexes were eluted and cross‐links were reversed at 65°C overnight. Samples were treated with RNase A (37°C, 30 min) and Proteinase K (55°C, 2 h). DNA was purified with spin columns and analysed by qPCR. Separate primer pairs were used to amplify the promoter regions containing R1, R2, and R3, allowing site‐region‐specific assessment of GLI1 enrichment. Relative enrichment was calculated by normalisation to input DNA, with normal rabbit IgG serving as the negative control. Primer sequences are listed in Table S5.

### 5.22 Statistical analysis

Statistical assessments were conducted using R (v 4.4.0) and appropriate packages and GraphPad Prism (v 8.0.1). For animal experiments, the individual mouse was the biological and statistical unit, and *n* denotes the number of mice per group. OARSI grades, osteophyte scores, and semiquantitative IHC scores were treated as ordinal outcomes. Prespecified two‐group comparisons were analysed using the Mann–Whitney *U* test, whereas comparisons involving more than two animal groups were analysed using the Kruskal–Wallis test followed by Dunn's multiple‐comparison test. Continuous mouse‐level molecular measurements were analysed using unpaired two‐tailed Student's *t*‐tests or nonparametric alternatives, as appropriate. Cell‐culture experiments were performed independently three times with a minimum of three biological replicates per condition. For CCK‐8 and CHX time‐course experiments, treatment and time were modelled jointly using two‐way ANOVA followed by multiplicity‐adjusted post hoc comparisons, rather than by a series of independent pairwise tests at individual time points. Paired comparisons of GLI1 or USP25 expression between grossly degenerated and relatively preserved cartilage from the same patient were performed using paired two‐tailed Student's *t*‐tests. The association between USP25 and GLI1 protein abundance across human cartilage specimens was evaluated using Spearman's correlation analysis. Other two‐group cellular comparisons were analysed using unpaired two‐tailed Student's *t*‐tests or Mann–Whitney *U* tests, as appropriate, and comparisons among more than two groups were analysed using one‐way ANOVA with the indicated post hoc procedure. The DUB screens were evaluated using the prespecified threshold‐based hit‐selection rules described above, and the two screening hit sets were integrated by prioritising candidates supported by both screening directions. Continuous data are presented as mean ± SD unless otherwise indicated; ordinal mouse outcomes are analysed at the individual‐mouse level and summarised using nonparametric statistics. Significance levels were denoted as *p < .05; **p < .01; ***p < .001; ****p < .0001; ns, not significant.

## AUTHOR CONTRIBUTIONS

*Conceptualisation*: DH and JL. *Experimental methods*: QZ, YY, and YH. *Analysis and interpretation of the data*: DH, JL, QZ, and YY. *Drafting of the manuscript*: QZ. *Critical revision and editing of the manuscript*: all the authors. *Funding acquisition*: JL and QZ.

## CONFLICT OF INTEREST STATEMENT

The authors declare no conflicts of interest.

## ETHICS STATEMENT

All patients have provided informed consent, and approved by the Seventh Affiliated Hospital of Sun Yat‐sen University (KY‐2024‐115‐01). All animal experimental protocols were approved by the Seventh Affiliated Hospital of Sun Yat‐sen University (No.SYSU‐IACUC‐2025‐B1057). Experimental procedures were conducted in strict accordance with the guidelines of the National Institutes of Health Guide for the Care and Use of Laboratory Animals. All authors agree to publish this manuscript.

## Supporting information



Supporting Information

Supporting Information

Supporting Information

Supporting Information

Supporting Information

Supporting Information

Supporting Information

Supporting Information

Supporting Information

## Data Availability

All public gene‐expression datasets used in this study are available from the Gene Expression Omnibus under the accession numbers listed in Table S1. Source data supporting the main quantitative results will be provided as supplementary source‐data files, including the relevant uncropped immunoblot images with corresponding lane and biological‐replicate annotations. For the public transcriptomic analyses, the processed data and key analysis information necessary to reproduce the principal results will also be provided as supplementary materials.
