# Supplementary material for: USP25 stabilises GLI1 through K48‐linked deubiquitination within a reciprocal regulatory circuit in osteoarthritis models
Source: Clin Transl Med. 2026 Sep 28;16(10):e70851. doi: 10.1002/ctm2.70851 (PMC13617533; doi:10.1002/ctm2.70851)
Supplement: Supplementary file 1 [file CTM2-16-e70851-s005.docx]

**Supplementary information**

**Supplementary Fig. S1. GLI1 was identified as a highly expressed gene of OA in the GSE51588 dataset. a** The outcomes of the PCA conducted on GSE51588. **b** Correlations between the 14 module eigengenes and OA. **c** Scale independence, mean connectivity, and scale-free topology graphs were generated with a soft threshold of 20. **d** Strong positive correlations were detected between GS and MM within the purple module (r = 0.62, *p = 1.0 x 10^-^*^5^). Points within the red rectangle were those with a GS > 0.5 and MM >0.8. **e** A volcano graphic illustrating OA-associated DEGs, with log_2_(fold-change) plotted against -log_10_(adjusted p-value). Red and blue points denote upregulated and downregulated DEGs, respectively, whereas genes exhibiting no significant expression differences are depicted in gray. **f** Based on the highly DEGs in (e), a PPI network was constructed using the STRING database, and 229 genes with a degree of ≥ 10 were obtained.

**Supplementary Fig. S2. Compartment-dependent GLI1 expression patterns across cartilage, synovium, and peripheral blood mononuclear cell datasets in OA. a-b** UMAP plots showing the distribution of cartilage-related samples from the GEO datasets before (a) and after (b) batch correction and integration. **c** Comparison of GLI1 expression between normal and OA cartilage samples in the integrated cartilage dataset, showing significantly higher GLI1 expression in OA cartilage. **d** GSEA identified differential Hedgehog-pathway enrichment between OA and normal cartilage in the batch-corrected integrated cartilage dataset comprising GSE178557, GSE129147, and GSE114007. **e** GLI1-stratified enrichment analysis in the batch-corrected integrated cartilage dataset comprising GSE178557, GSE129147, and GSE114007. **f-g** UMAP plots showing the distribution of synovial tissue samples from the GEO datasets before (f) and after (g) batch correction and integration. **h** Comparison of GLI1 expression between normal and OA synovial tissue samples in the integrated synovial tissue dataset, showing significantly lower GLI1 expression in OA synovial tissue. **i** GSEA identified differential Hedgehog-pathway enrichment between OA and normal synovial tissue in the batch-corrected integrated synovial tissue dataset comprising GSE1919, GSE12021, GSE55235, and GSE55457. **j** GLI1-stratified enrichment analysis in the batch-corrected integrated synovial dataset comprising GSE1919, GSE12021, GSE55235, and GSE55457. **k** Comparison of GLI1 expression between normal and OA peripheral blood mononuclear cell samples from the GSE300310 dataset, showing no significant difference between the two groups. **l** GSEA showed no significant difference in Hedgehog-pathway enrichment between OA and normal peripheral blood mononuclear cell samples in GSE300310. **m** GLI1-stratified enrichment analysis likewise showed no significant difference between OA and normal peripheral blood mononuclear cell samples in GSE300310.

**Supplementary Fig. S3. Complementary validation of USP25 prioritization as a GLI1-regulating deubiquitinase.** **a** Representative immunoblots from the loss-of-function DUB screen showing GLI1 protein abundance after individual DUB depletion in SW1353 cells treated with IL-1β (10 ng/mL for 24 h); the screen was repeated independently three times. **b-d** Spearman correlations between *USP25* mRNA and *BMI1*, *MYC*, and *BCL2* expression in GSE51588. **e-f** RT-qPCR analysis of *Bmi1*, *Myc*, and *Bcl2* in differentiated ATDC5 cells treated with IL-1β (10 ng/mL for 24 h) after USP25 overexpression or shUSP25-mediated depletion. Data are presented as mean ± SD from three independent experiments with at least three biological replicates per condition and were analyzed using unpaired two-tailed Student’s t-tests. **g** Immunoblotting of Hedgehog-pathway components in the same ATDC5 conditions; representative blots from three independent experiments are shown. Significance levels were denoted as **p < 0.01; ***p < 0.001; ****p < 0.0001; ns, not significant.

**Supplementary Fig. S4. a** Representative immunofluorescence images of USP25 and GLI1 distributions in SW1353 cells and differentiated ATDC5 cells treated with IL-1β (10 ng/mL for 24 h) after the indicated constructs. Fluorescence-signal overlap was quantified using PCC and thresholded Manders’ coefficients (M1 and M2). For each cell model and each independent experiment, 10 cells randomly selected from multiple non-overlapping fields were analyzed, and the mean value of the 10 cells was used as one experiment-level value. Data are presented as mean ± SD from three independent experiments. **b** Reciprocal Co-IP in HEK293T cells transfected with vector, USP25, or GLI1 constructs. **c** Reciprocal Co-IP in SW1353 cells and differentiated ATDC5 cells treated with IL-1β (10 ng/mL for 24 h) after the indicated USP25 or GLI1 constructs. **d** Immunoblotting of GLI1 and USP25 in SW1353 cells and differentiated ATDC5 cells expressing the catalytically inactive USP25^C178A^ mutant. **e** HEK293T cells with USP25 perturbation were treated with MG132 (10 μM) or chloroquine (50 μM) for 6 h before immunoblotting of USP25 and GLI1. Representative biochemical assays from three independent experiments are shown.

**Supplementary Fig. S5. Functional effects of USP25 perturbation in SW1353 cells and differentiated ATDC5 cells treated with IL-1β (10 ng/mL for 24 h). a-f** RT-qPCR and **g-h** immunoblotting of MMP9, MMP13, and COL2A1 after shUSP25-mediated depletion, USP25 overexpression, or USP25C178A expression. **i-l** CCK-8 assessment of cellular metabolic viability under the corresponding conditions. **m-n** Representative images and quantification of EdU incorporation as a DNA-synthesis readout. Cell experiments were performed independently three times with at least three biological replicates per condition. Two-group endpoint comparisons were analyzed using unpaired two-tailed Student’s t-tests, and CCK-8 time-course data were analyzed using two-way ANOVA with multiplicity-adjusted post hoc comparisons. Scale bar: 400 μm.

**Supplementary Fig. S6. Schematic illustration of animal experiments, created with BioRender. a** Diagram of the nine animal groups and study timeline. Groups 1-2 were C57BL/6J (WT) mice receiving intra-articular shUSP25. Groups 3-6 were *Usp25^fl/fl^*; *Col2a1*-Cre^ERT^+ mice treated with corn-oil vehicle and designated *Usp25*-ctrl; Groups 7-9 were mice from the same *Usp25^fl/fl^*; *Col2a1*-Cre^ERT^+ conditional line treated with tamoxifen (30 mg/kg/day for 5 consecutive days) to induce conditional *Usp25* recombination and designated *Usp25*-cKO. Corn oil or tamoxifen was administered at week 0, sham or DMM surgery at week 2, treadmill exercise from week 3 for 4 weeks, intra-articular intervention from week 7 once weekly for 2 weeks, and euthanasia at week 9. **b** The conditional line was generated by breeding *Usp25^fl/fl^* mice with *Col2α1*-Cre^ERT^ transgenic mice. *Usp25*-cKO mice were produced by administering tamoxifen (30 mg/kg/day for 5 consecutive days) intraperitoneally to *Usp25*-ctrl mice for 5 days. **c** DMM surgery combined with treadmill exercise was used to induce osteoarthritis, whereas sham surgery served as the control. Each animal group contained n = 6 mice. This figure is schematic.

**Supplementary Fig. S7. Schematic illustration of the design for animal experiments, created with BioRender. a** Comparison of USP25 depletion achieved by intra-articular shUSP25 and tamoxifen-induced conditional recombination (Groups 1 vs 7 under sham and Groups 2 vs 8 under DMM). **b** Association between the tamoxifen-induced *Col2a1*-Cre^ERT^-mediated *Usp25*-depletion state and DMM-related cartilage pathology relative to the corn-oil-treated *Usp25*-ctrl state (Groups 3 vs 7 under sham and Groups 4 vs 8 under DMM). **c** GLI1 rescue analysis using Groups 4, 5, 8, and 9, with Group 8 vs Group 9 as the principal rescue contrast. **d** AZ1-associated activity in DMM-operated *Usp25*-ctrl mice (Group 4 vs Group 6). Each animal group contained n = 6 mice. This figure is schematic.

**Supplementary Fig. Graphical Abstract. GLI1-centered USP25-GLI1 reciprocal regulation in OA-relevant models.** Hedgehog/GLI1 signaling transcriptionally reinforces USP25 expression, while USP25 deubiquitinates and stabilizes GLI1. This reciprocal circuit is associated with inflammatory and cartilage-catabolic changes in the tested models. AZ1, a reported dual USP25/USP28 inhibitor, is presented as a proof-of-concept pharmacological probe; the AZ1-associated phenotype observed in this study was partly USP25 dependent.
