# Supplementary material for: USP25 stabilises GLI1 through K48‐linked deubiquitination within a reciprocal regulatory circuit in osteoarthritis models
Source: Clin Transl Med. 2026 Sep 28;16(10):e70851. doi: 10.1002/ctm2.70851 (PMC13617533; doi:10.1002/ctm2.70851)
Supplement: Supplementary file 2 [file CTM2-16-e70851-s009.docx]

**Supplementary Table S1. The detailed information of the GEO datasets used for analysis in this study**

| **GEO dataset** | **Platform** | **Species** | **Tissue** | **OA** | **Normal** | **Country** | **PMID** |
| --- | --- | --- | --- | --- | --- | --- | --- |
| GSE51588 | GPL13497 | Homo sapiens | Subchondral bone | 40 | 10 | USA | 24229462 |
| GSE178557 | GPL13497 | Homo sapiens | Knee cartilage | 4 | 4 | China | 35733354 |
| GSE114007 | GPL11154 | Homo sapiens | Knee cartilage | 10 | 8 | USA | 30081074 |
|  | GPL18573 | Homo sapiens | Knee cartilage | 10 | 10 |  |  |
| GSE129147 | GPL15207 | Homo sapiens | Knee cartilage | 10 | 9 | Turkey | 31142155 |
| GSE1919 | GPL91 | Homo sapiens | Synovial tissues | 5 | 5 | Germany | 20858714 |
| GSE12021 | GPL96 | Homo sapiens | Synovial tissues | 10 | 9 | Germany | 18721452 |
| GSE55235 | GPL96 | Homo sapiens | Synovial tissues | 10 | 10 | Germany | 24690414 |
| GSE55457 | GPL96 | Homo sapiens | Synovial tissues | 10 | 10 | Germany | 24690414 |
| GSE300310 | GPL24676 | Homo sapiens | Peripheral blood mononuclear cells | 6 | 6 | China | - |

**Supplementary Table S2. Information for antibodies used in this study**

| **Protein** | **Assay** | **Catalog number** | **Company** |
| --- | --- | --- | --- |
| *β-Tubulin* | WB | Ab52623 | Abcam |
| *GAPDH* | WB | Ab181602 | Abcam |
| *GLI1* | WB;IF;IHC | Ab289368 | Abcam |
| *USP25* | WB;IF;IHC | Ab246948 | Abcam |
| *USP28* | WB | Ab126604 | Abcam |
| *Ptch1* | WB | Ab53715 | Abcam |
| *Ptch2* | WB | Ab151775 | Abcam |
| *Smo* | WB | Ab235183 | Abcam |
| *Kif7* | WB | Ab95884 | Abcam |
| *SuFu* | WB | Ab28083 | Abcam |
| *BMI1* | WB | Ab126783 | Abcam |
| *MYC* | WB | Ab32072 | Abcam |
| *BCL2* | WB | Ab117115 | Abcam |
| *GLI2* | WB | Ab277800 | Abcam |
| *Flag* | WB | Ab205606 | Abcam |
| *Ub* | WB | Ab134953 | Abcam |
| *HA* | WB | Ab236632 | Abcam |
| *MMP9* | WB;IHC | Ab76003 | Abcam |
| *MMP13* | WB;IHC | Ab39012 | Abcam |
| *COL2A1* | WB;IHC | Ab307674 | Abcam |

**Supplementary Table S3. Primers for quantitative PCR**

| **Primer set** | | **Primers** | **Sequence (5’-3’)** | **Product size (bp)** |
| --- | --- | --- | --- | --- |
| SW1353/  HEK293T/  Primary chondrocytes | *GLI1* | Forward  Reverse | 5'- CCCAGACAGAGGCCCAC -3'  5'- GAGGTGCGGATAACCGTCTG -3' | 123 |
|  | *BMI1* | Forward  Reverse | 5'- CGCCCTTTGTTGAGAGGATTTT -3'  5'- TGCTGGTCTCCAGGTAACGA -3' | 186 |
|  | *MYC* | Forward  Reverse | 5'- TGGAAAACCAGCCTCCCG -3'  5'- TTCTCCTCCTCGTCGCAGTA -3' | 112 |
|  | *BCL2* | Forward  Reverse | 5'- GATAACGGAGGCTGGGATGC -3'  5'- TGACTTCACTTGTGGCCCAG -3' | 155 |
|  | *USP25* | Forward  Reverse | 5'- CAGCAAGCCTTGAAGGATAGTA -3'  5'- TCATTGCCAGGAAGTGCTGT -3' | 125 |
|  | *MMP9* | Forward  Reverse | 5'- TTCTGCCCGGACCAAGGATA -3'  5'- GACCATAGAGGTGCCGGATG -3' | 175 |
|  | *MMP13* | Forward  Reverse | 5'- CAGTTTGCAGAGCGCTACCT -3'  5'- TTCTCGGAGCCTCTCAGTCA -3' | 102 |
|  | *COL2A1* | Forward  Reverse | 5'- CATGAGGGCGCGGTAGAG -3'  5'- TGCCAGCCTCCTGGACAT -3' | 192 |
| ATDC5 | *GLI1* | Forward  Reverse | 5'- GGTCTCGGGGTCTCAAACTG -3'  5'- CGGCTGACTGTGTAAGCAGA -3' | 133 |
|  | *BMI1* | Forward  Reverse | 5'- TGCTGGAGAGCTGGAAAGTG -3'  5'- GTGAGGGAACTGTGGGTGAG -3' | 130 |
|  | *MYC* | Forward  Reverse | 5'- GGTGTCTGTGGAGAAGAGGC -3'  5'- TTGTGCTGGTGAGTGGAGAC -3' | 132 |
|  | *BCL2* | Forward  Reverse | 5'- GAGTTCGGTGGGGTCATGTG -3'  5'- CTGGGGCCATATAGTTCCACAA -3' | 161 |
|  | *USP25* | Forward  Reverse | 5'- CAGTTTTGCAGTCGGCAATTA -3'  5'- TTTGGTGCCTGGTTCTGGAT -3' | 130 |
|  | *MMP9* | Forward  Reverse | 5'- AAACCTCCAACCTCACGGAC -3'  5'- CTGAAGCATCAGCAAAGCCG -3' | 122 |
|  | *MMP13* | Forward  Reverse | 5'- GACCCCAACCCTAAGCATCC -3'  5'- CCTCGGAGACTGGTAATGGC -3' | 80 |
|  | *COL2A1* | Forward  Reverse | 5'- GCCAGGATGCCCGAAAATTAG -3'  5'- ACGATCACCTCTGGGTCCTT -3' | 131 |

**Supplementary Table S4. Targeting sequences for shRNAs**

| **shRNAs** | | **Sequences** |
| --- | --- | --- |
| SW1353/  HEK293T/  Primary chondrocytes | sh*USP25*#1 | 5'- GCACTTCTCCTGTTGACGATA -3' |
|  | sh*USP25*#2 | 5'- GCTGTAGAAGATATGAGAAAT -3' |
|  | sh*USP28*#1 | 5'- GCACAGAAGTTCGTTGTCATA -3' |
|  | sh*USP28*#2 | 5'- GCTCTAATGATGGGATCAAAT -3' |
|  | sh*GLI1*#1 | 5'- CCTGATTATCTTCCTTCAGAA -3' |
|  | sh*GLI1*#2 | 5'- CGTGAGCCTGAATCTGTGTAT -3' |
| ATDC5 | sh*USP25*#1 | 5'- CGGTGGATGAAGTATAATGAT -3' |
|  | sh*USP25*#2 | 5'- CCTGCTGGTTTAGTGCAGTTA -3' |
|  | sh*GLI1*#1 | 5'- CCTATCCTGATCCACCAGAAA -3' |
|  | sh*GLI1*#2 | 5'- CCTGTGTACCACATGACTCTA -3' |

**Supplementary Table S5. Primers for ChIP-qPCR**

| **Primer set** | **Primers** | **Sequence (5’-3’)** | **Product size (bp)** |
| --- | --- | --- | --- |
| USP25-Region1 | Forward  Reverse | 5'- TGCTTAATTTGGCAACTGGGAT -3'  5'- TGTGTCAGTCACTGCTAGGC -3' | 150 |
| USP25-Region2 | Forward  Reverse | 5'- GAGAGAACGCGTGACACAGA -3'  5'- TTCCCTCCATGCCCATAACG -3' | 77 |
| USP25-Region3 | Forward  Reverse | 5'- CCAAGCTTGCTTTCCTTGTCA -3'  5'- GAGGTGAGAGTGCAGCAGTG -3' | 116 |
